# New fossil cichlid from the middle Miocene of East Africa revealed as oldest known member of the Oreochromini

**DOI:** 10.1038/s41598-019-46392-5

**Published:** 2019-07-15

**Authors:** Stefanie B. R. Penk, Melanie Altner, Alexander F. Cerwenka, Ulrich K. Schliewen, Bettina Reichenbacher

**Affiliations:** 1 Department of Earth and Environmental Sciences, Ludwig-Maximilians-Universität München, 80333 Munich, Germany; 2 GeoBio-Center, Ludwig-Maximilians-Universität München, 80333 Munich, Germany; 3 Department of Ichthyology, SNSB Bavarian State Collection of Zoology, 81247 Munich, Germany; 4 Section Evertebrata varia, SNSB Bavarian State Collection of Zoology, 81247 Munich, Germany

**Keywords:** Palaeontology, Environmental sciences

## Abstract

A new genus and species of fossil cichlid fishes of middle Miocene age (12.5 Ma) is described from the Ngorora fish *Lagerstätte* (Tugen Hills, Kenya) in the East African Rift Valley. Parsimony analysis of morphological characters using published phylogenetic frameworks for extant cichlids combined with the application of a comprehensive best-fit approach based on morphology was employed to place the new fossil taxon in the phylogenetic context of the African cichlids. The data reveal that the fossil specimens can be assigned to the tribe Oreochromini within the haplotilapiines. †*Oreochromimos kabchorensis* gen. et sp. nov. shows a mosaic set of characters bearing many similarities to the almost pan-African *Oreochromis* and the East African lake-endemic *Alcolapia*. As the striking diversity of present-day African cichlids, with 1100 recognised species, has remained largely invisible in the fossil record, the material described here adds significantly to our knowledge of the Miocene diversity of the group. It effectively doubles the age of a fossil calibration point, which has hitherto been used to calibrate divergence times of the East African cichlids in molecular phylogenetic investigations. Furthermore, the comparative dataset derived from extant cichlids presented here will greatly facilitate the classification of fossil cichlids in future studies.

## Introduction

Cichlid fishes (Cichliformes) represent one of the most diverse vertebrate families, comprising about 220 genera and over 1700 recognised species^1,2^. They are widely distributed in tropical freshwater environments, with some species entering brackish or alkaline habitats (e.g.^3–5^). Their evolutionary success has been attributed to numerous morphological and behavioural adaptations, such as the occurrence of both oral and pharyngeal jaws with specialised dentition, diverse mating systems, different modes of parental care (e.g. mouthbrooding), and visual sensitivity to nuptial male colouration (see^6–8^). Cichlidae have been classified into four subfamilies, i.e. the Etroplinae (limited to Madagascar, Sri Lanka and India), the Ptychochrominae (restricted to Madagascar), the Cichlinae (found only in the Neotropics), and the Pseudocrenilabrinae (widely distributed in Africa and the Middle East)^9–14^. In light of their current geographic distribution and their phylogenetic relationships, the first cichlids could have emerged in the Late Jurassic or Early Cretaceous, i.e. prior to the fragmentation of Gondwana (e.g.^13,15–18^). On the other hand, recent palaeontological and molecular-clock-based time calibrations indicate a Late Cretaceous or early Cenozoic date of origin^19–22^.

**Figure 1:**
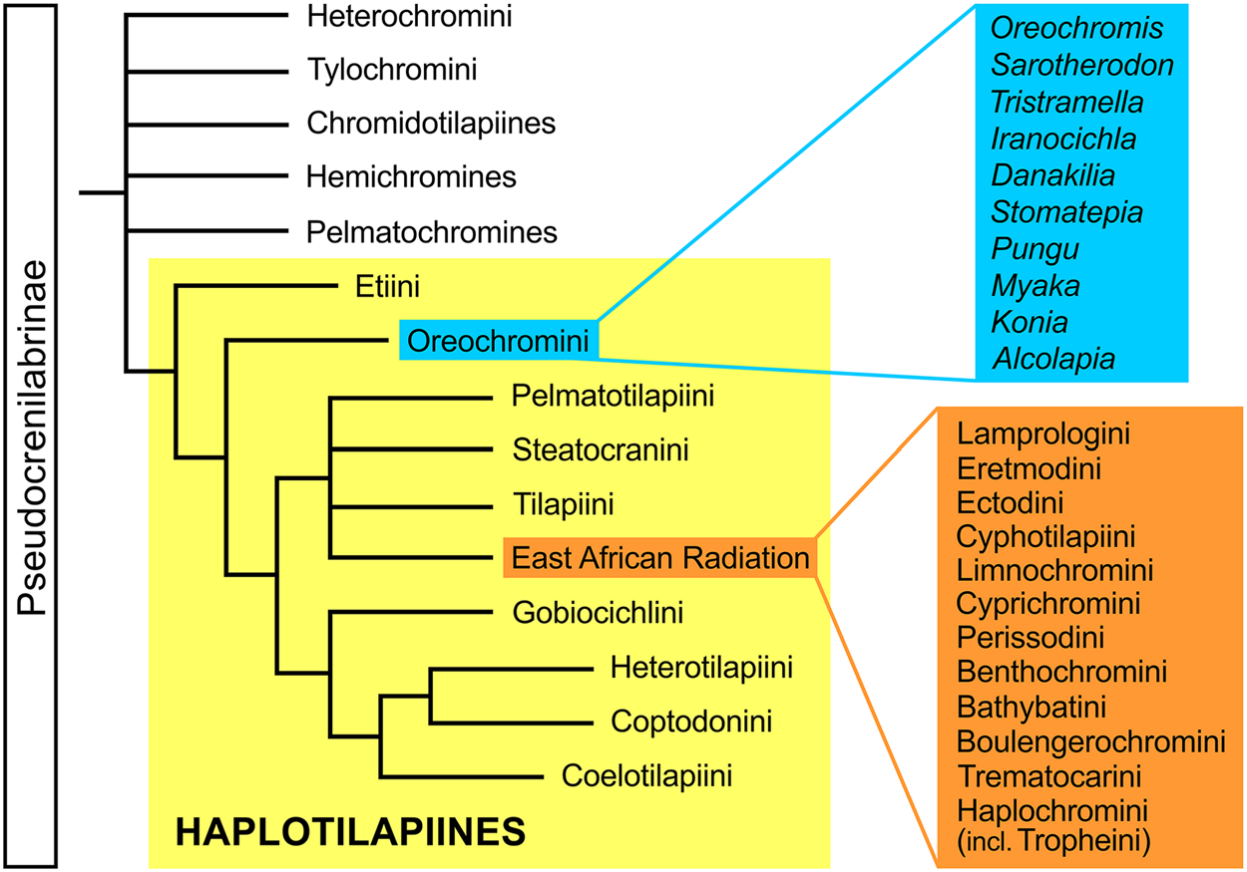
Simplified composite tree showing recently proposed phylogenetic relationships among the non-haplotilapiine Pseudocrenilabrinae and the haplotilapiines (yellow box). In addition, the names of the genera belonging to the Oreochromini (blue box) and the names of the tribes involved in the East African Radiation (orange box) are listed. Source of tree topology: Altner *et al*.^37^; source of phylogenetic data: refs^25,36,60^.

Of the four cichlid subfamilies, the Pseudocrenilabrinae (African and Middle East cichlids) form the largest clade, with approximately 150 genera and 1100 species (e.g.^1,23^). Within the Pseudocrenilabrinae, the haplotilapiines constitute the most diverse subclade (refs^24,25^; Fig. 1). While extant haplotilapiines can be clearly defined by molecular genetics, their members share only one morphological apomorphy, namely the presence of tricuspid inner teeth in the oral jaws^24^. Haplotilapiine cichlids are divided into 22 tribes (Fig. 1). Thirteen of these represent the clade that encompasses the members of the Lake Tanganyika radiation, also termed the East African Radiation (EAR), which includes the numerous species endemic to the Great Lakes of the East African Rift Valley (Tanganyika, Malawi, Victoria) and some riverine species^7,25–27^. Among the nine halotilapiine tribes that did not contribute to the EAR, the Oreochromini are particularly important, because their extant representatives display an essentially pan-African distribution, with a few additional species in the Middle East^28^. Some of these are “widespread/riverine” genera (*Oreochromis*, *Sarotherodon*, *Tristramella*, *Iranocichla*, *Danakilia*), while others are “lake-endemics” (*Stomatepia*, *Pungu*, *Myaka*, *Konia*, *Alcolapia*)^29,30^.

## Brief Historical Review of The Concept of Haplotilapiine Tribes

The first approach to classifying the extant African and Levantine cichlid species was developed by Regan^31,32^. He recognised two major complexes, namely the *Tilapia* and *Haplochromis* groups, based on differences in the composition of the pharyngeal apophysis. Over half a century later, Greenwood^33^ re-investigated the same structure in more detail and discerned a further subdivision, leading to the recognition of four “types” of apophysis (named for *Tylochromis*, *Tilapia*, *Tropheus*, *Haplochromis*). In addition, he asserted that phylogenetic interrelationships cannot be reconstructed on the basis of the structure of the pharyngeal apophysis. Trewavas^28^, however, followed Regan^31,32^ in distinguishing two groups, and raised each to the level of a tribe (Tilapiini and Haplochromini). Subsequently, Poll^34^ used meristic and morphometric data, scales, dentition, soft-tissue anatomy, and osteology to define a total of 12 tribes for the cichlids endemic to Lake Tanganyika (LT). The concept of tribes employed by Poll^34^ was later revised and expanded by Takahashi^35^, who concluded that 16 tribes are present in LT, each of which is characterised by a particular combination of internal and external morphological characters. In addition, Takahashi^35^ was the first to propose a morphologically based phylogeny for the LT tribes. This phylogeny is, in part, supported by recent molecular genetic analyses^7,25–27,36^.

### The contribution of fossil specimens to cichlid phylogeny

One of the main challenges in understanding the evolutionary history of cichlids is to establish robust fossil-based calibration points. This is partly due to the scanty fossil record of the Cichlidae, especially when compared with the high species diversity of the family’s extant representatives. Only 35 fossil cichlid species have been reported based on articulated skeletons, with ages ranging from the Eocene to the Pliocene of North and East Africa, South America, Haiti, Saudi Arabia, and Europe (e.g.^5,21,37–42^). Further fossil cichlid remains have been described on the basis of isolated bones and teeth, but most of these were not identifiable at genus and species level (e.g.^5,43–47^). The second major problem is that attribution of fossil cichlids to extant genera and tribes is often impossible. This is due to difficulties related to (i) taphonomy, (ii) the generally conservative “bauplan” of cichlids and the frequent occurrence of convergent evolution, (iii) the scarcity of comprehensive surveys of skeleton-related characters (those most likely to be preserved in fossils), and (iv) the dearth of phylogenetic matrices that include skeletal traits.

(i) The taphonomic problems arise because even in the rare cases when fossil skeletons are well preserved, informative characters may be lost, damaged or otherwise be unrecognisable (e.g. lacrimal morphology, composition of caudal skeleton) (see^47^).
(ii) The generally conservative nature of the “bauplan” of cichlid fishes means that relatively few morphological characters may differentiate between species, genera, and even tribes (see^14^). Furthermore, convergence and parallel evolution can result in similarities between cichlid species and genera that are only distantly related^48,49^. All these factors can greatly complicate the systematic assignment of cichlids, and the problems are exacerbated when only characters expressed in hard parts are available, as is generally the case for fossils (see^50^).
(iii) Important studies have been conducted on the morphology and variation of hard parts (teeth, scales, bones) within and between extant cichlid species. However, these reports have either focused on a specific character group, such as scales (e.g.^51–53^) or infraorbital bones (e.g.^54^), or concentrated on a particular cichlid group (e.g.^28,34,55–59^). Additional morphological information can be found by searching the literature, but no truly comprehensive survey has yet been compiled. Furthermore, care is necessary regarding the assignment of a species to a certain tribe in previous studies because the composition of tribes may have been revised in the light of more recent molecular phylogenetic work (see^60^).
(iv) Character matrices for the phylogenetic analysis of cichlids based on morphological characters have been published by^14,35,58^. Most of the characters, however, relate to soft tissue or delicate bony structures that are seldom preserved in a fossil. This is probably the main reason why these matrices have not yet been used to locate any fossil cichlid in its phylogenetic context.

### The “best-fit approach”

The “best-fit approach” applied in our work assumes that each extant cichlid tribe is characterised (and can be recognised) by a certain combination of characters rather than by one or very few autapomorphies. To implement the “best-fit approach”, it is crucial to determine which of the extant cichlid genera or tribes exhibit the combination of characters that occurs in the fossil. In this respect the “best-fit approach” conforms to the established taxonomic assignment of fossil taxa. The salient difference lies in the nature of the comparative dataset of extant species that serves as the basis for the taxonomic assignment of the fossil. In the case of the “best-fit approach”, this dataset includes representatives of **all** extant lineages and **all** extant genera to which the fossil could in principle belong. For the present study, this information was obtained from an extensive survey of the literature on extant cichlids and by assembling a comprehensive comparative dataset for extant cichlid species (see Suppl. Data 3, Tables S1–S8 and Materials and Methods for details). The new data acquired in this way is available in the Suppl. Tables S9–S14, which can now serve as an unprecedented source of data for future systematic placements of cichlid fossils. Pending the establishment of new phylogenetic datasets that can be applied to fossil cichlids, the “best-fit approach” is currently the most practical method for reliably assigning a cichlid fossil at the level of genus and tribe. Although not explicitly referred to as such, it has recently been used in two studies on fossil cichlids from the same *Lagerstätte* in Kenya as the material described here^37,39^. It resulted in the identification of **†***Tugenchromis pickfordi* as the first fossil representative of the Lake Tanganyika radiation^37^ and the recognition of †*Rebekkachromis ngororus* and †*R*. *kiptalami* as the oldest known fossil members of the haplotilapiines, with possible affinities to the tribe Etiini^39^. Meanwhile, the availability of **†***T*. *pickfordi* as a new fossil calibration point has led to the currently most precise estimate (13.7–12.7 Ma) for the date of origin of the Lake Tanganyika radiation^20^, which highlights the importance of attributing fossil cichlids at higher systematic levels as precisely as possible.

The objective of this study is to present newly discovered, very well-preserved cichlid fish fossils from the middle Miocene (12.5 Ma) of Kenya (East Africa) and, by addressing the aforementioned problems directly, to determine whether these fossils represent an extant lineage or tribe. Our work builds on the phylogenetic frameworks constructed by^14,35^ and the application of the “best-fit approach”.

### Study site

**Figure 2:**
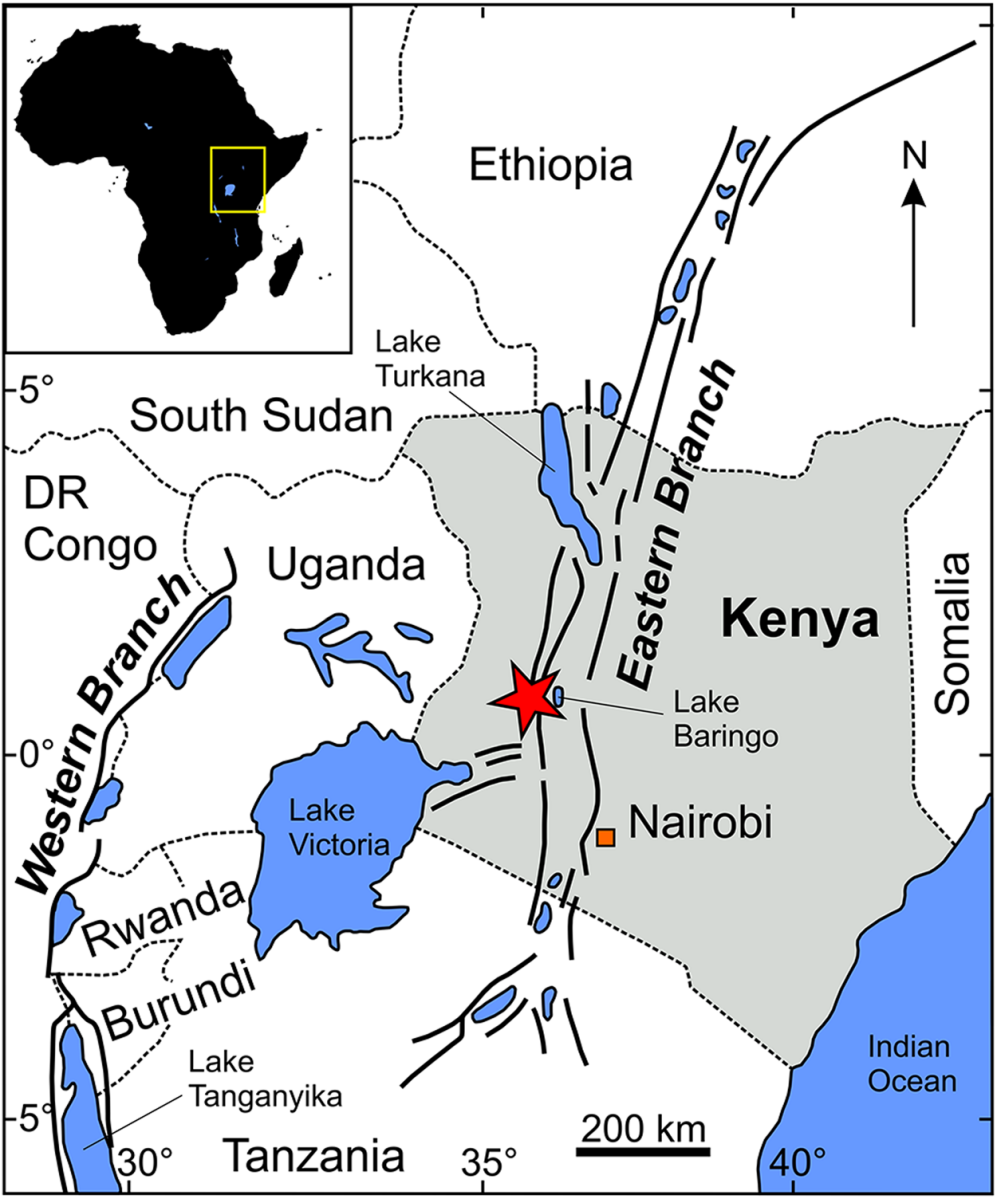
Geographical map of Eastern Africa illustrating the Western and Eastern branches of the East African Rift System. The red star marks the location of the study area, the Tugen Hills (Baringo County, Central Kenya Rift). Map reprinted from Kiage & Liu^110^ (slightly modified), with permission from Elsevier.

The study area is located within the Central Kenya Rift, which is part of the Eastern branch of the East African Rift System (see^61^) (Fig. 2). The specimens were collected in the year 2013 in the Tugen Hills (see Fig. 2 in Rasmussen *et al*.^62^) at the sites Kabchore-2a and -2c (GPS coordinates 0°46′10.13″N, 35°48′6.16″E and 0°46′5.99″N, 35°48′10.73″E). Both sites belong to the lowermost part of Member C of the Ngorora Formation and can be assigned to the middle Miocene (ca. 12.5 Ma) based on lithostratigraphic criteria and correlation^62^. The fossil-yielding sediments are fine-laminated marls. Apart from further articulated fish fossils, isolated fish remains and a few leaves were found.

### Systematic palaeontology

Family **Cichlidae** Bonaparte, 1835

Subfamily **Pseudocrenilabrinae** Fowler, 1934

Tribe **Oreochromini** Dunz & Schliewen, 2013

**†*Oreochromimos kabchorensis*** gen. et sp. nov.

(Figs 3–5).

**Figure 3:**
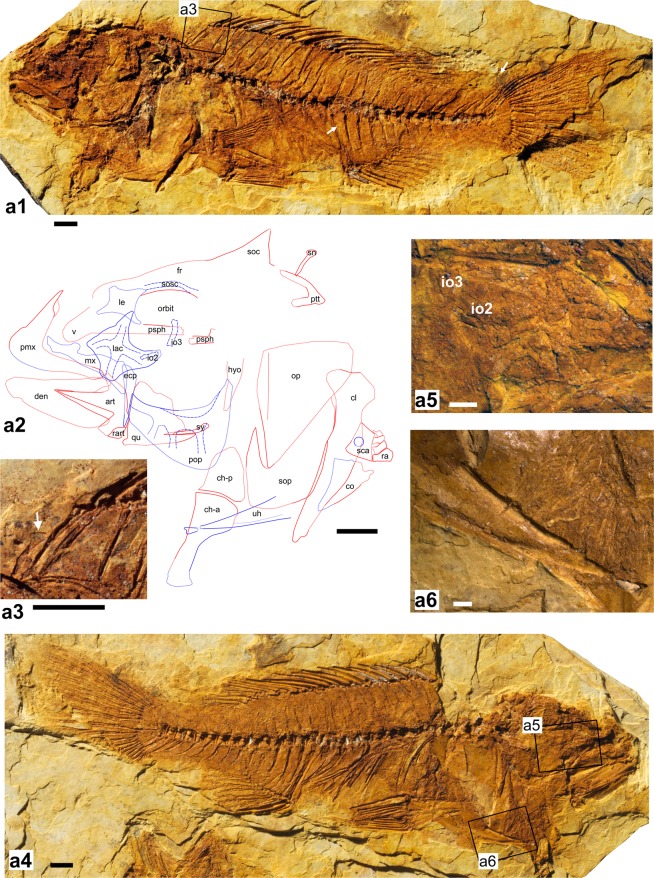
Holotype of †*Oreochromimos kabchorensis* gen. et sp. nov., OCO-2c-1a, b(1). (**a1**) Articulated skeleton of part, arrows indicates end of longest dorsal fin ray and first tubular scale of the posterior lateral line segment, respectively. **(a2)** Interpretative drawing of the head; red and blue lines indicate bones best recognisable on part (**a1**) and counterpart (**a4**), respectively; dotted lines indicate tentative outline due to preservation. (**a3**) Close-up of the predorsal region showing imprint of supraneural bone (arrow). (**a4**) Articulated skeleton of counterpart. (**a5**) Close-up of lacrimal bone and infraorbital bones 2 and 3. **(a6)** Close-up of urohyal bone. Scale bars: 5 mm (**a1**–**a4**), 1 mm (**a5**–**a6**). Photos of a1 and a4 by M. Schellenberger at the SNSB - Bavarian State Collection of Palaeontology and Geology (BSPG). Abbreviations: art, angulo-articular; ch-a, anterior ceratohyal; ch-p, posterior ceratohyal; cl, cleithrum; co, coracoid; den, dentary; ecp, ectopterygoid; fr, frontal; io, infraorbital; lac, lacrimal; le, lateral ethmoid; mx, maxilla; op, opercle; pmx, premaxilla; pop, preopercle; psph, parasphenoid; ptt, posttemporal; qu, quadrate; ra, radial; rart, retro-articular; sca, scapula; sn, supraneural bone; soc, supraoccipital crest; sop, subopercle; sosc, supraorbital canal; sy, symplectic; uh, urohyal; v, vomer.

**Figure 4:**
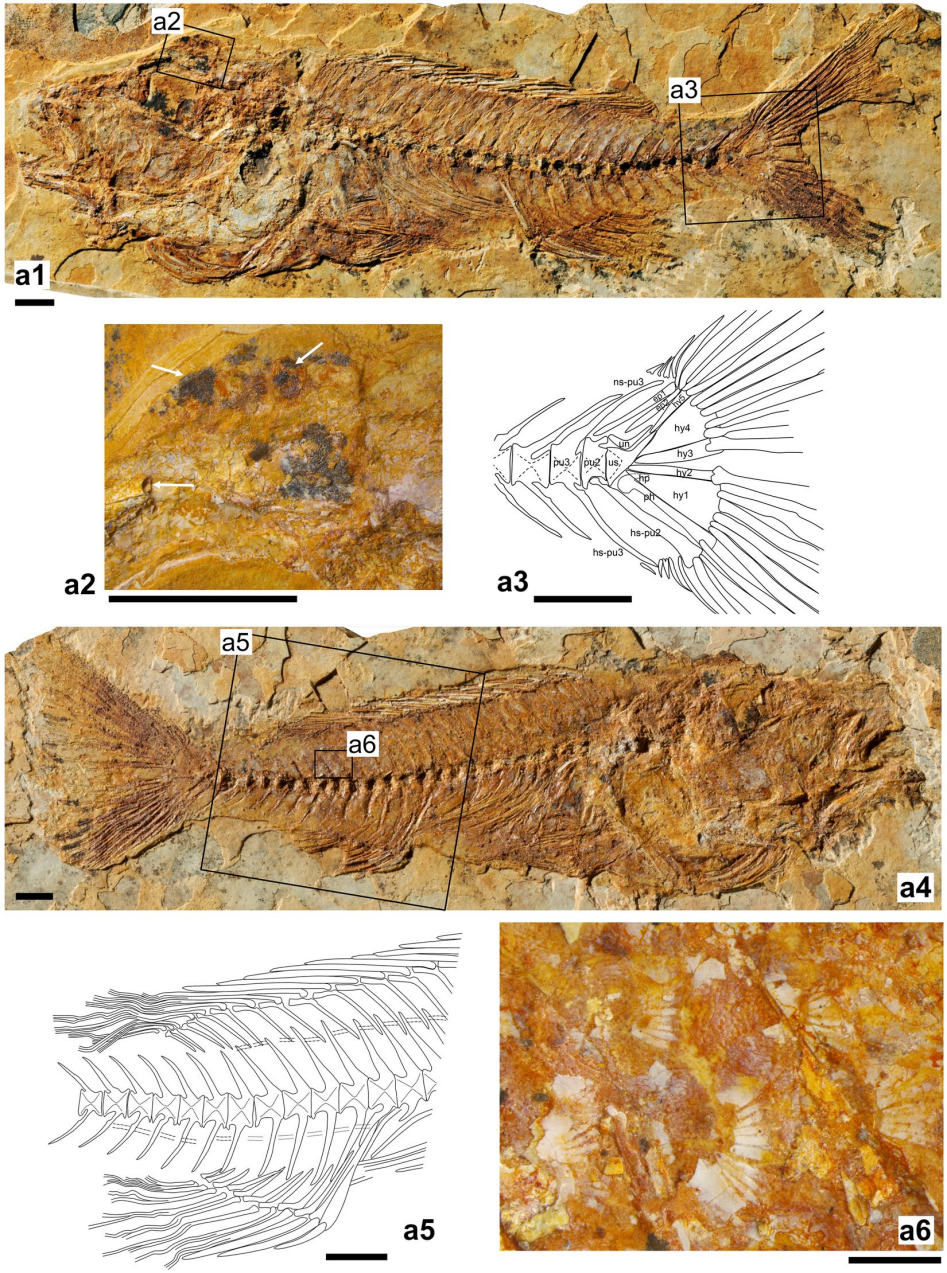
**(a1–a6)** Paratype OCO-2a-10a, b of †*Oreochromimos kabchorensis* gen. et sp. nov. (**a1, a4**) Articulated skeleton of part and counterpart. (**a2**) Close-up of the neurocranium showing the putative nuchal hump, scales, and the supraorbital sensory canal (all indicated with arrows). (**a3**) Reconstruction of the caudal skeleton, dotted lines indicate tentative outline due to preservation. (**a5**) Reconstruction of the posterior lateral line segment (complemented based on holotype). **(a6)** Flank scales visible between the neural spines beneath the soft rayed part of the dorsal fin. Scale bars: 5 mm (**a1**–**a5**), 1 mm (**a6**). Photos of a1 and a4 by M. Schellenberger at the SNSB - Bavarian State Collection of Palaeontology and Geology (BSPG). Abbreviations: ep, epural; hp, hypurapophysis; hs, haemal spine; hy, hypural plate; ns, neural spine; ph, parhypural; pu, preural vertebra; un, uroneural; us, urostyle.

**Figure 5:**
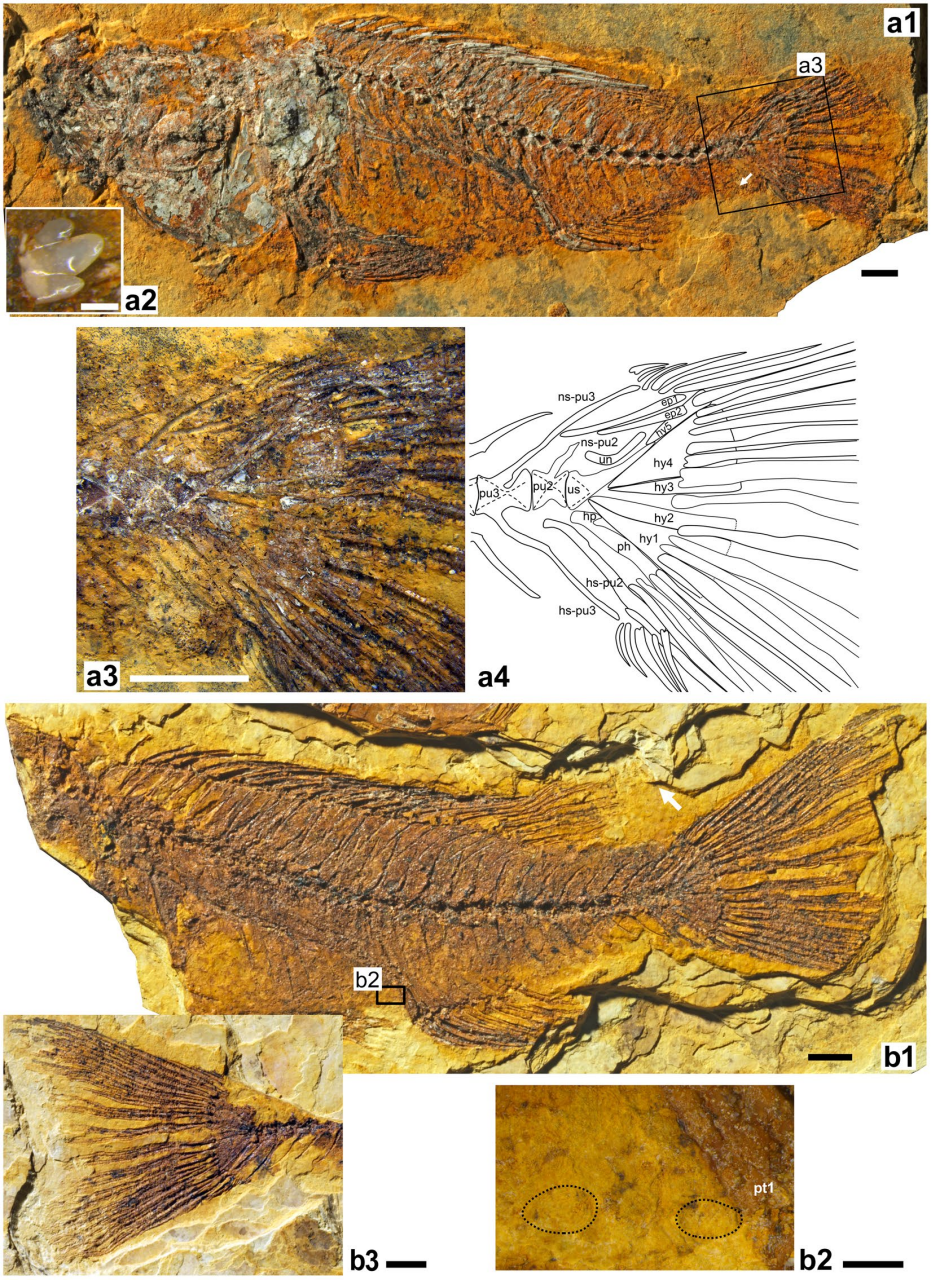
Paratypes of †*Oreochromimos kabchorensis* gen. et sp. nov. **(a1–a4)** Specimen OCO-2c-4a. **(a1)** Articulated skeleton, arrow indicates end of longest anal fin ray. **(a2)** Tricuspid dentary tooth preserved *in situ*. **(a3–a4)** Close-up of the caudal skeleton showing typical caudal fin arrangement, although fin rays are slightly displaced due to taphonomy (photo and reconstruction, dotted lines indicate uncertain outline due to preservation). (**b1–b3**) Specimen OCO-2c-1a, b(3). **(b1)** Incomplete articulated skeleton of part, arrow indicates end of longest dorsal fin ray. **(b2)** Close-up of belly scales between pelvic and anal fin. **(b3)** Caudal fin of counterpart displaying slightly emarginate shape. Scale bars 5 mm (**a1**,**a3**,**b1**,**b3**), 1 mm (**b2**), 0.1 mm (**a2**). Photos of (**a1**,**b1**,**b3**) by M. Schellenberger at the SNSB - Bavarian State Collection of Palaeontology and Geology (BSPG). Abbreviations: ep, epural; hp, hypurapophysis; hs, haemal spine; hy, hypural plate; ns, neural spine; ph, parhypural; pt1, first anal fin pterygiophore; pu, preural vertebra; un, uroneural; us, urostyle.

#### Generic diagnosis

Lacrimal bone with lateral line branched into four tubules, overlapped by second infraorbital bone; one club-shaped supraneural bone; absence of notch on cleithrum (*sensu* Murray & Stewart)^63^; proximally slender urohyal, probably without anterodorsal projection; ascending process of premaxilla shorter than horizontal ramus; dorsal process of angulo-articular curved; minute belly scales (*sensu* Seegers & Tichy)^29^. This character combination is not known from any other fossil or extant cichlid genus.

#### Type locality and stratigraphy

Kabchore, site 2a (0°46′10.13″N, 35°48′6.16″E) and 2c (0°46′5.99″N, 35°48′10.73″E), Tugen Hills, Central Kenya; Member C of the Ngorora Formation, ca. 12.5 Ma.

#### Etymology

“Oreochro”- derived from the extant cichlid genus *Oreochromis*; used to emphasize its close affinity to the tribe Oreochromini. The ancient Greek word “-mimos” (μῖμος) means “imitator”. Gender masculine. The specific name “*kabchorensis*” refers to the name of the type locality.

#### Holotype

OCO-2c-1a, b(1); complete skeleton preserved in part and counterpart; total length (TL) 13.4 cm, standard length (SL) 10.9 cm, and body length (BL) 6.8 cm (Fig. 3a1, a4).

#### Paratypes

Three complete skeletons preserved in part and counterpart (OCO-2c-4a, b, OCO-2a-10a, b, OCO-2c-13a, b), four incomplete skeletons lacking portions of the head, caudal fin endoskeleton, or anal fin (OCO-2a-5, OCO-2a-13(1), OCO-2c-1a, b(3), OCO-2c-5a, b(1)).

#### Species diagnosis

Same as for genus.

#### General description

Total length up to 13.4 cm, standard length up to 10.9 cm, body length up to 7.6 cm (Suppl. Data 3, Table S15), greatest body depth at dorsal fin origin. Rather stout, fusiform body, with moderately long and narrow caudal peduncle. Massive skull, putative nuchal hump on forehead, terminal snout, isognathous jaws, oral jaw dentition with unicuspid teeth (it is not possible to determine if these are from the outer or inner row(s)) and tricuspid inner teeth. Cycloid scales on head, body, pectoral fin base, and caudal fin; divided lateral line.

#### Neurocranium and infraorbital series

Forehead bulged, with small depression posteriorly. Two of the paratypes (OCO-2c-5a, b(1), OCO-2a-10a, b) exhibit a few scale rows above forehead that might indicate a nuchal hump (Fig. 4a2). Slightly curved frontal bone, anterior portion comparatively narrow, posterior part broad. Parietal bones not preserved. Supraoccipital crest triangular and moderately deep, posterior margin slightly concave. Sphenotic with anteroventral process. Supraorbital sensory canal on frontal bone running parallel to dorsal margin of orbit (Figs 3a2 and 4a2). Parasphenoid long and straight; broad vomer recognisable. Lateral ethmoid approximately square and relatively large (Fig. 3a2). Mesethmoid with ovate posterior portion. Small, tube-like remains of nasal bones apparent close to ascending premaxillary process. The holotype has a partially preserved otolith, but no taxonomically useful structures are preserved.

Infraorbital series comprises at least three infraorbital bones (Fig. 3a2, a5); lacrimal (=first infraorbital) bearing four, relatively broad tubules of the lateral line (Figs 3a2, a5); lacrimal shape approximately square with straight to concave margins; lacrimal depth (*sensu* Trewavas)^28^ 12% of head length. Canals on infraorbitals 2–3 more or less elongated tubes, second infraorbital overlaps lacrimal at its posterodorsal edge.

#### Oral jaws and teeth

Premaxilla robust, head slightly protruding and rounded; horizontal ramus longer than ascending process; articular process not well preserved (Fig. 3a2). Maxilla incompletely preserved, approximately as long as premaxilla, head with two robust processes, rest of the bone straight with rounded end. Dentary robust and anteriorly truncated, its lower arm longer and deeper than upper arm, lateral face of lower arm bears at least three relatively large ovate lateral line foramina. Angulo-articular triangular, slightly longer than deep; posterior margin with small facet for lateral condyle of quadrate; dorsal process curved anteriorly (Fig. 3a2). Roughly triangular retro-articular bone well preserved.

**Figure 6:**
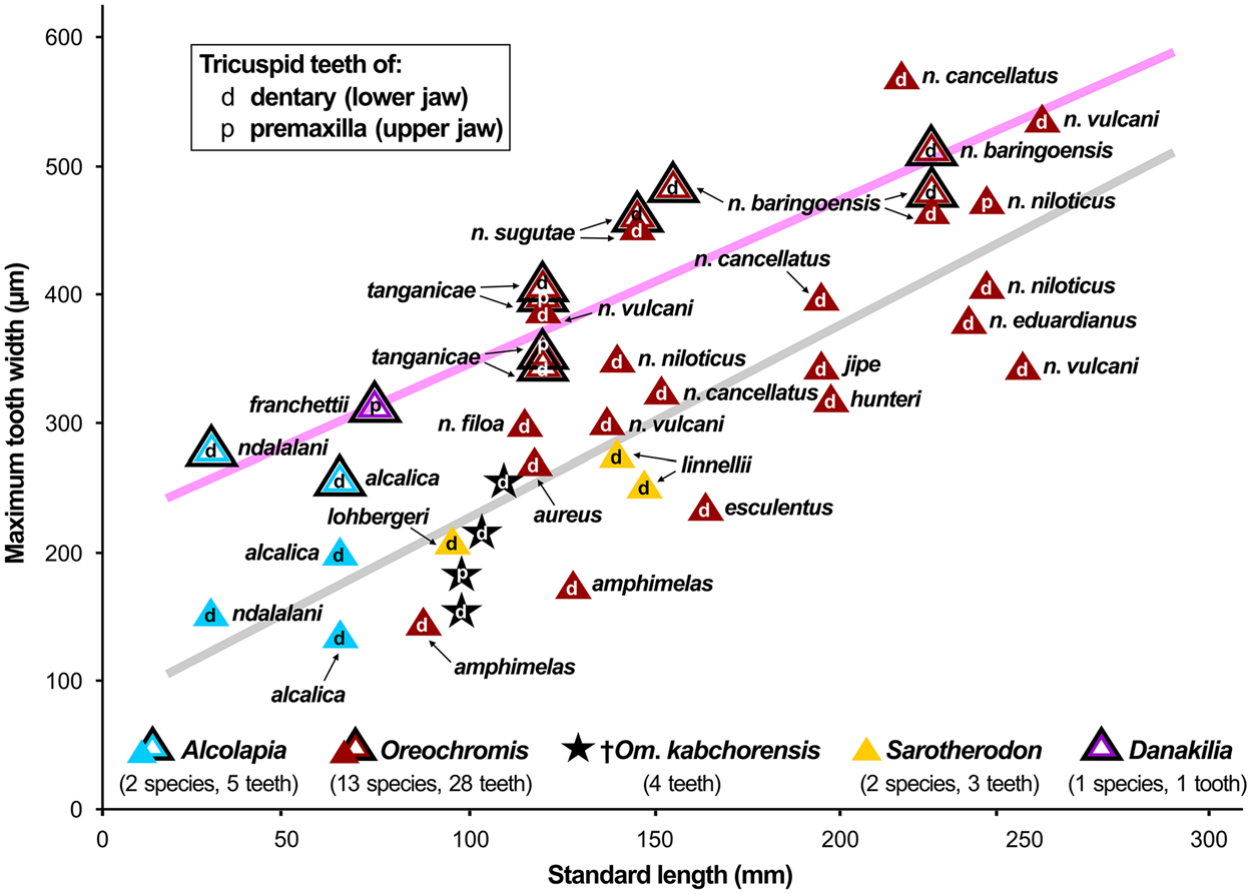
Maximum crown widths of tricuspid oral teeth of †*Oreochromimos kabchorensis* gen. et sp. nov. (this study) and recent species of *Alcolapia*, *Oreochromis*, *Sarotherodon*, and *Danakilia* compiled from the literature (refs^28,95^ for *Oreochromis*, *Sarotherodon*, and *Danakilia*; Tichy & Seegers^80^ for *Alcolapia*). Outer-row teeth (n = 11) indicated with unfilled triangles in a black frame, inner-row teeth (n = 30) indicated with filled triangles; colours indicate taxa. Trend line for outer-row teeth in pink, trend line for inner-row teeth in grey. Abbreviation: *n*. for *niloticus*.

A total of four small tricuspid teeth (maximum crown width 0.15–0.26 mm) could be clearly discerned; each tooth presents a comparatively large median tip and small flanking cusps (Fig. 5a2). Three of these tricuspid teeth (one tooth each) were preserved *in situ* in the anteriormost region of the premaxilla (OCO-2a-10b) and in the anteriormost region of the dentary (holotype, paratype OCO-2c-4a). The fourth tricuspid tooth was found isolated in the paratype OCO-2a-10b; its presence immediately next to the dentary indicates that it represents a lower jaw tooth. The measurements of the teeth (black stars in Fig. 6) plot near those of the tricuspid inner teeth of *Oreochromis* and *Sarotherodon* specimens that had similar body sizes to the fossil specimens (see Suppl. Data 3, Table S16 for data). Further teeth of unicuspid conical shape were found in the specimens close to the premaxilla (OCO-2a-10b; OCO-2c-13a) or as imprints in the anteriormost region of the dentary (holotype; OCO-2c-4a; OCO-2c-13b).

#### Suspensorium and opercular apparatus

Symplectic elongate and pointed anteriorly. Quadrate triangular, with rounded dorsal margin; anterior margin nearly straight, articulating with ectopterygoid; preopercular process elongate and pointed posteriorly, bearing a hook-shaped lateral condyle; anterior margin and preopercular process form an angle of 76°. Ectopterygoid prominent, tapering ventrally. Entopterygoid not well preserved. Dorsal portion of palatine strongly curved. Opercle with convex to straight anterior and oblique posteroventral margin, ventrally with slightly rounded tip, posterodorsal margin slightly angular; suspensoriad ridge (*sensu* Barel *et al*.^64^) parallel to anterior margin. Subopercle with prominent pointed process in front of opercular ventral corner. Preopercle robust, crescent-shaped, four lateral line foramina recognisable ventrally (visible in holotype); horizontal limb broad; vertical limb incomplete, latter slightly narrower.

#### Hyoid and branchial arches

Left and right hyoid bars, urohyal, and remains of ten branchiostegal rays (five on each side) recognisable. Anterior and posterior ceratohyals not in contact, latter bone slightly tapering (Fig. 3a2). Anterior ceratohyal elongate and robust, broadened posteriorly; anterior margin with posteroventrally directed urohyad spine. Urohyal preserved in lateral view, slightly V-shaped (Fig. 3a6); condyle relatively small; anterior shaft elongate; probably no anterodorsal spine or projection (if a small spine had been present it cannot be excluded that it was broken off). Gill filaments are recognisable in the region of the opercular bones in most specimens (Fig. 3a4).

#### Vertebral column

Vertebral column slightly curved; 28–30 vertebrae, of which 13–15 are abdominal (Suppl. Data 3, Table S15). Vertebral centra hourglass-shaped, first and penultimate centra relatively shorter than all others. Compared to the caudal vertebrae, abdominal vertebrae have more massive neural arches and more inclined neural spines. Caudal vertebrae generally with equally sized neural and haemal spines, haemal spine of first caudal vertebra located posterior to first anal fin pterygiophore. 11–13 rib pairs, first pair on third vertebra, ribs long and slender, parapophyses increasing in length in posterior direction. Epineural bones recognisable at height of first vertebra to eleventh rib pairs. Supraneural bone proximally slender and markedly angled and thickened distally (=club-shaped, Fig. 3a3); usually slightly displaced.

#### Pectoral girdle and fins

Posttemporal, cleithrum, dorsal and ventral postcleithra, scapula, and coracoid discernible. Complete posttemporal bone visible in holotype (Fig. 3a2) and specimen OCO-2a-10a, b, forked, with two relatively straight and relatively slender processes, dorsal process longer than ventral one. Cleithrum very robust, with a prominent longitudinal ridge; proximal part of cleithrum broad and extended, no notch (*sensu* Murray & Stewart^63^) recognisable (Fig. 3a2), distal part comparatively slender. Dorsal postcleithrum large and broad, tapering distally, posterior margin convex, anterior margin almost straight (visible in OCO-2c-4b). Ventral postcleithrum stout, revealing a long, distally pointed portion. Scapula rectangular with a large foramen, coracoid triangular (Fig. 3a1, a2).

Pectoral fin supported by four small, roughly hourglass-shaped radials that increase in size dorsally to ventrally (Fig. 3a2); number of rays 12–13 (Suppl. Data 3, Table S15); rays are completely preserved in holotype (Fig. 3a1) and do not extend to the anal fin origin.

#### Pelvic girdle and fins

Pelvic bones insert beneath pectoral fins, preserved in ventral view (Figs 3a1, a4, 4a1, a4, 5a1). Basipterygia elongate and triangular, each with a subpelvic external keel, a moderately long accessory subpelvic keel, a narrow anterior process, and a short posterior process at the inner margin. Each pelvic fin has one strong spine and five branched, segmented rays; rays do not reach anal fin origin (Fig. 3a1, a4).

#### Dorsal fin

Dorsal fin continuous, 13–14 spines +9–11 branched, segmented rays (Suppl. Data 3, Table S15). Lengths of spines increase posteriorly, last spine being about three to four times the length of the first. Rays do not reach posterior margin of hypural plates (Figs 3a4 and 5b1). 22–24 stout pterygiophores; distal end of first pterygiophore shows prominent, anteriorly directed protrusion; pterygiophore of last dorsal fin spine inserts behind neural spine of penultimate or last abdominal vertebra (i.e. vertebra 12, 13 or 14); pterygiophores that support rays decrease in size backwards, with the posteriormost one supporting two rays.

#### Anal fin

Anal fin with 3 strong spines +8–10 branched, segmented rays (Suppl. Data 3, Table S15). Spines increasing in length, with last spine being about twice as long as first. Specimen OCO-2c-4a shows that longest ray extends to penultimate vertebra (Fig. 5a1); in all other specimens rays visible only up to middle of caudal peduncle. As usual in cichlid fishes, the first two anal fin spines are supported by a robust, relatively long pterygiophore composed of two fused elements; it inserts before the first caudal vertebra (Fig. 4a5). Last spine and rays (except last two rays) each supported by pterygiophores, decreasing in size backwards; last pterygiophore supports two rays.

#### Caudal endoskeleton and fin

The caudal fin is fan-shaped and slightly emarginate at its end (Fig. 5b3). Sixteen segmented principal rays can be discerned, eight (seven branched and one unbranched) in each lobe (Figs 4a3 and 5a3, a4, b1, b3). The procurrent rays are unbranched, their number is six dorsally and six to seven ventrally (Figs 4a3 and 5a4; Suppl. Data 3, Table S15). The caudal endoskeleton consists of five autogenous hypural plates, an autogenous parhypural, an autogenous uroneural, the urostyle, two preural vertebra, and two slender epurals (Figs 4a3 and 5a4). All hypural plates are separated by thin sutures and a relatively narrow, elongate diastema separates hypural plates 2 and 3. Hypural plates 1 and 4 are the largest hypural plates, hypural plate 5 is small (Figs 4a3 and 5a4). The long and distally slightly broader parhypural is placed close to hypural plate 1 and the urostyle, but is not connected to the latter. It bears a posteriorly directed hypurapophysis (Figs 4a3 and 5a4). The urostyle forms the complement for the uroneural bone, which is positioned between hypural plate 5 and epural 2. The preural centrum 2 either has a neural arch without a neural spine (Fig. 4a3) or a reduced neural spine (Fig. 5a4), and an autogenous haemal arch with a well-developed spine that broadens distally. The preural centrum 3 has fully developed neural and haemal arches with complete spines that extend to the procurrent rays (Figs 4a3 and 5a4).

#### Squamation

Scales cycloid and variable in sizes and shapes. Large scales with 8–12 radii and mostly continuous circuli occur on the flank, caudal peduncle, and caudal fin base; a few have disintegrated circuli in the caudal field (Fig. 4a6). Further large scales (albeit slightly smaller than those just mentioned) cover cheek (which is fully scaled), opercle, subopercle, and interopercle; opercle with two vertical rows of scales running along the anterior margin and one horizontal row along the dorsal margin. Lacrimal bone, together with dorsal, anal, and pelvic fins scaleless. Very small and ovate scales on putative nuchal hump (Fig. 4a2), chest, pectoral-fin base, and belly (Fig. 5b2); very small and elongate scales present at dorsal and ventral margins of caudal peduncle. Scales are also found on the proximal two-thirds of the caudal fin. These are disposed in different configurations: (i) A few vertical scale rows, each comprising up to four very small and elongate scales, occur in the gap between the upper and lower caudal fin lobes; (ii) two horizontal rows with very small elongate scales are visible in the space between the segmented parts of the caudal fin rays; (iii) one horizontal row with intermediate-sized scales cover the space between the non-segmented parts of the caudal fin rays.

Lateral line divided into two segments along the body. Two scale rows between anterior lateral line segment and vertebral column (body axis *sensu* Takahashi^35^). Anterior segment almost completely preserved in paratype OCO-2a-10a, b, bearing probably 17 tubular scales that bridge the tips of the neural spines and extend to the fifth caudal vertebra. Posterior segment always incompletely preserved, but the holotype and paratype OCO-2a-10a, b reveal different parts of it. It starts at the level of the first caudal vertebra, overlaps the proximal to middle parts of the haemal spines, and is covered by at least 10 tubular scales (Fig. 4a5).

### Phylogenetic analysis

Stiassny^14^ and Takahashi^35^ are two important sources of phylogenetic matrices that included African cichlids and were based on morphological characters. We used Stiassny^14^ to analyse the position of the new fossil among all Cichlidae, and Takahashi^35,54^ to investigate its position within the haplotilapiines. The new fossil was added to the respective morphological data matrices by coding character states for six out of 28 characters in the matrix based on Stiassny^14^ (see Suppl. Data 1 for nexus file and Suppl. Data 3, Table S17 for characters and states) and by utilizing 15 out of 41 characters in the matrix based on Takahashi^35,54^ (see Suppl. Data 2 for nexus file and Suppl. Data 3, Table S18 for characters and states). Additional details are provided in the Methods section.

**Figure 7:**
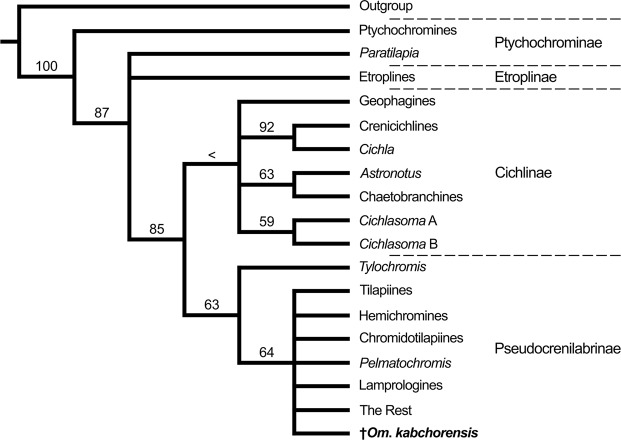
Phylogenetic interrelationships of 17 cichlid ingroup taxa representing the four known subfamilies currently recognised (Etroplinae, Ptychochrominae, Cichlinae, Pseudocrenilabrinae) and the phylogenetic position of †*Oreochromimos kabchorensis* gen. et sp. nov. (highlighted in bold). This is the strict consensus tree derived from the two most parsimonious trees (MPTs) produced by TNT from the modified morphological data matrix of Stiassny^14^. Tree length (TL) = 35 steps, consistency index (CI) = 0.89, retention index (RI) = 0.94. Bootstrap values from 1000 pseudoreplicates are presented on the branches (values below 50% indicated with “<”).

Maximum-parsimony analysis of the morphological data matrix based on Stiassny^14^ plus the new fossil taxon resulted in two most parsimonious trees (MPTs) and a strict consensus tree (Fig. 7). The topology of the latter is congruent with Stiassny’s^14^ phylogeny. Stiassny’s “ptychochromines” is resolved with maximum bootstrap support as the most basal group within the Cichlidae, while *Paratilapia* forms a polytomy with her “etroplines”. The “etroplines” along with *Paratilapia* are reconstructed as being sister to the highly supported Cichlinae + Pseudocrenilabrinae clade. Cichlinae and Pseudocrenilabrinae are each resolved as monophyletic. †*Oreochromimos kabchorensis* gen. et sp. nov. is placed in the clade of the Pseudocrenilabrinae. It is recovered as being nested within a moderately supported polytomy that comprises all African cichlids except *Tylochromis*. It should be noted that the systematic concept employed by Stiassny^14^ is not fully congruent with the current understanding of the subfamilies. Her “ptychochromines” comprised two members of the Ptychochrominae (*Ptychochromoides*, *Ptychochromis*), but not *Paratilapia*, which is also a member of this subfamily according to Sparks & Smith^13^. Furthermore, Stiassny’s “etroplines” included *Etroplus*, *Paretroplus*, and *Oxylapia*. While the first two correspond to the subfamily Etroplinae, molecular studies have recognised *Oxylapia* as a member of the Ptychochrominae (e.g.^13^). However, as †*Oreochromimos kabchorensis* is clearly assigned to the Pseudocrenilabrinae, this discrepancy has no impact on its placement.

**Figure 8:**
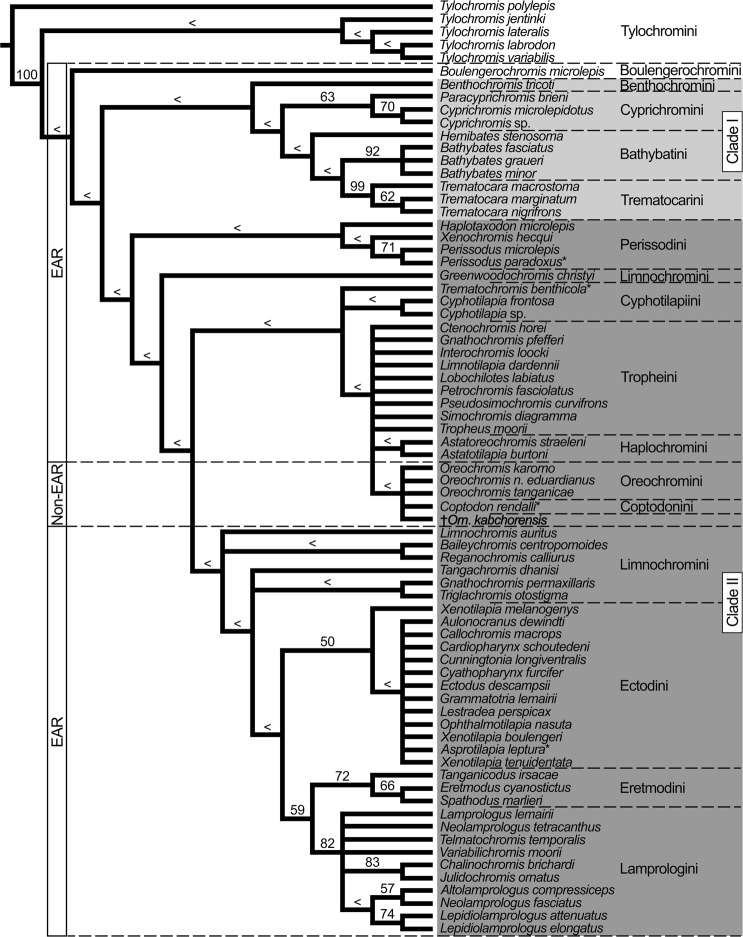
Phylogenetic interrelationships of 67 haplotilapiine ingroup species from Lake Tanganyika and the phylogenetic position of †*Oreochromimos kabchorensis* gen. et sp. nov. (highlighted in bold). This is the strict consensus tree derived from the six MPTs produced by TNT from the modified morphological data matrix of Takahashi^35^. TL = 197 steps, CI = 0.42, RI = 0.79. Bootstrap values from 1000 pseudoreplicates are presented on the branches (values below 50% indicated with “<”). Superscript asterisks refer to updated names (based on Catalog of Fishes, Eschmeyer *et al*.^104^). The valid names and the corresponding names used by Takahashi^35^ are as follows: *Asprotilapia leptura* (as *Xenotilapia*), *Coptodon rendalli* (as *Tilapia*), *Perissodus paradoxus* (as *Plecodus*), and *Trematochromis benthicola* (as *Ctenochromis*). Abbreviations: EAR, East African Radiation; *n*., *niloticus*.

Maximum-parsimony analysis of the morphological data matrix based on Takahashi^35,54^ plus the new fossil taxon yielded six MPTs and a strict consensus tree (Fig. 8). The topology of the latter is largely consistent with the strict consensus tree published by Takahashi^35^. The monotypic tribe Boulengerochromini is recovered as the most basally diverging lineage among those members of the haplotilapiines examined. It is resolved as being sister to two major clades. With respect to the content of these two clades in our tree and that derived by Takahashi^35^, the only difference is that the Ectodini are nested within the second major clade in our phylogeny, but in the first major clade in the phylogeny of Takahashi^35^. Most of the tribes are resolved as being monophyletic; the exceptions are Bathybatini, Limnochromini, Cyphotilapiini, and Oreochromini. Trematocarini is resolved with almost maximum bootstrap support of 99% and Lamprologini and Eretmodini are each recovered with strong bootstrap support of 82% and 72%, respectively. Monophyly of Tropheini, Haplochromini, Oreochromini + Coptodonini, as well as most of the remaining clades is supported with bootstrap support below 50%. †*Oreochromimos kabchorensis* gen. et sp. nov. is nested within the Oreochromini + Coptodonini clade, with a bootstrap support below 50%. We believe that the meagre support for the clades in both parsimony analyses (based on refs^14,35,54^) reflects the large number of missing data in the case of our fossil. It should be mentioned that some differences exist between the terminologies of the tribes used in Takahashi^35^ and subsequent publications. While Takahashi^35^ considered Trematocarini as a junior synonym of Bathybatini, we follow Weiss *et al*.^36^ in recognising Trematocarini as a separate clade. “Greenwoodochromini” in Takahashi^35^ are now part of the Limnochromini^65^ and the “New tribe” represented by *Trematochromis benthicola* in Takahashi^35^ belongs to the Cyphotilapiini (e.g.^36^).

## Discussion

### Systematic assignment of †*Oreochromimos* gen. nov

Cichlids are well supported as a monophyletic group by recent molecular phylogenetic studies (e.g.^66,67^). Their monophyly is also recognised on the basis of several morphological synapomorphies summarized by Casciotta & Arratia^50,68^. Among them are soft tissue characters such as separate A_2_ and A_w_ sections of the adductor mandibulae complex (Stiassny^69^), or an extendible blind pouch of the stomach (Zihler^70^), or delicate structures like the presence of short, paired hypapophyses on anterior vertebrae (character 77 in Kullander^58^), or an anterocaudal pseudocolliculum in sagittal otoliths (Gaemers^71^) (see also^50,68^, and references therein). Unfortunately, none of these soft-tissue characters or delicate structures have a good chance of being preserved in a fossil (see^49^). Therefore it is not surprising that they are not discernible in our fossil specimens. Identifiable otoliths are also not preserved. Nevertheless, the new fossil taxon reveals the following diagnostic features which, taken together, are specific to modern cichlid fishes (see^68,72–75^): (i) it exhibits an interrupted lateral line, which is typical for Cichlidae but comparatively rare among extant fishes^76^; (ii) the caudal endoskeleton comprises five autogenous hypural plates, two epural bones, an autogenous uroneural, an autogenous parhypural bone with a hypurapophysis at its proximal end, a preural centrum 2 bearing an autogenous haemal spine and a neural arch with no or a reduced neural spine, and a preural centrum 3 that is fused with its haemal spine; (iii) eight principal caudal fin rays occur in each lobe. The attribution of the fossil to the Cichlidae is additionally supported by a single dorsal fin bearing several spines and rays, five branchiostegal rays on each hyoid bar, and a pelvic-fin formula of one spine and five rays (see^10,77^). Furthermore, the dorsal and anal fin ray counts (Suppl. Data 3, Table S15), as well as the total number of recognisable lateral-line scales lie within the range for extant Cichlidae (see^78^).

### Assignment to the Pseudocrenilabrinae

Morphological characters that define the Pseudocrenilabrinae (without *Heterochromis*) comprise delicate configurations of muscles and ligaments (e.g. characters 22 and 26 in Cichocki^10^: *adductor arcus palatini* muscle inserting into the palatine fossa extending onto the palatine, anteroventral palatomaxillary ligament present and originating proximally on the maxillary process of the palatine). Further defining characters, amongst others, are a first epibranchial bone with an elongated uncinate process (character 24 in Stiassny^14^) and the presence of a strongly pigmented opercular spot (character 26 in Stiassny^14^) (see also ref.^58^, characters 1, 24, 52–53, 61, and 90). As written above for the cichlid’ synapomorphies, also these characters have a very limited fossilization potential, if any, and are not present in our fossil specimens. However, assignment of the new fossil cichlid to the Pseudocrenilabrinae is supported by the result of our phylogenetic analysis (Fig. 7). Finally, it appears rather unlikely that Cichlinae, which are now endemic to the Neotropics, should be represented with a single species in middle Miocene sediments in East Africa.

### Assignment to the haplotilapiines

Most of the extant representatives of the Pseudocrenilabrinae are characterised by an oral dentition comprised of an outer row of teeth and one or more rows of inner teeth; each row can bear uni-, bi-, and/or tricuspid teeth, and a distinct downsizing trend is seen from the outer towards the inner row(s) (e.g.^34,35,59,60,79–81^). Among the Pseudocrenilabrinae, only the haplotilapiines are characterised by the occurrence of tricuspid teeth in the inner row of the oral jaw dentition; indeed, this is the only known morphological autapomorphy for this molecularly well-supported clade^24^. While the detection of inner- and outer-row teeth is simple in recent species, it turns out to be more difficult in fossil specimens, and is possible only in the case of very well-preserved fossils. Examples are described in^39,47,82^. †*Oreochromimos kabchorensis* gen. et sp. nov. has small tricuspid oral teeth that can be interpreted as inner-row teeth (Fig. 6). Accordingly, the new fossil taxon can be recognised as a member of the haplotilapiines.

### Assignment at the level of tribe

In the following, the terms “EAR tribes” and “non-EAR tribes” are used to differentiate between the set of tribes that have contributed to the East African Radiation (EAR) and those that have not. In our phylogenetic analysis, which is based on Takahashi^35,54^, †*Om*. *kabchorensis* gen. et sp. nov. is placed within the non-EAR tribes Oreochromini and Coptodonini (Fig. 8). However, the taxon sampling used by Takahashi is not representative for the haplotilapiines in general because it is restricted to tribes occurring in Lake Tanganyika. This explains why Oreochromini and Coptodonini (both non-EAR) are deeply nested within the EAR tribes in the tree shown in Fig. 8. Accordingly, the position of †*Om*. *kabchorensis* in that tree must be treated with caution, and we turned to the “best-fit approach” as the next step. We postulate that †*Om*. *kabchorensis* can be assigned to that cichlid tribe with which it shares all character states without exception. Consequently, we set out to identify th(os)e tribe(s) with which the new fossil taxon reveals a 100% fit in its fossilised characters.

**Figure 9:**
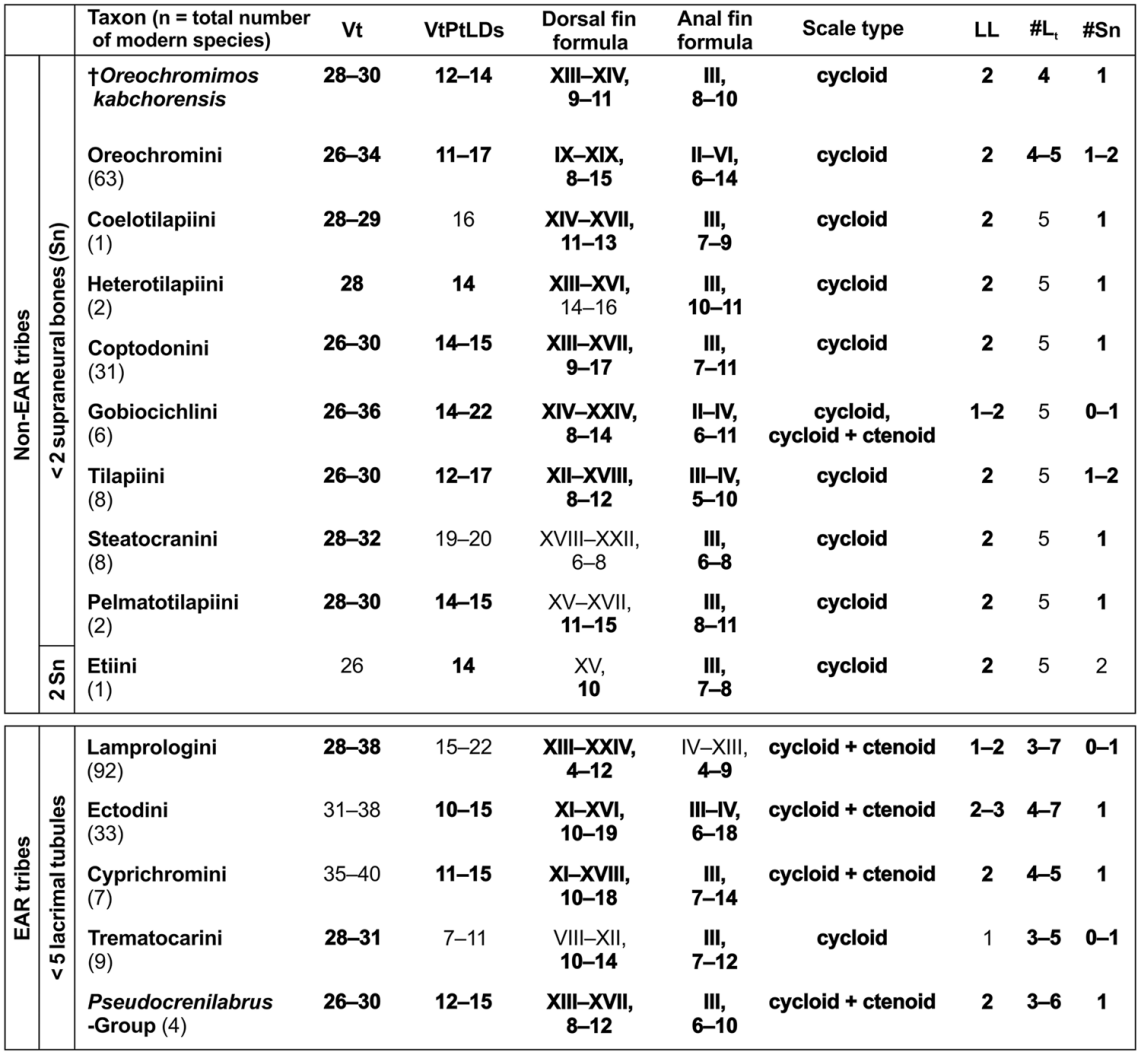
Morphological characters (ranges) of all modern species of the non-EAR tribes, of those EAR tribes in which <5 lacrimal tubules can occur (lineage *Pseudocrenilabrus* Group included in the tribe Haplochromini), and of †*Oreochromimos kabchorensis* gen. et sp. nov. Total numbers of species for each tribe were compiled from the literature (see Suppl. Data 3, Table S6, S8). Numbers of total vertebrae (Vt, including urostyle), dorsal/anal fin formulas, scale types, lateral line segments (LL), lacrimal tubules (#L_t_), and numbers of supraneural bones (#Sn) are from this study and from literature (see Suppl. Data 3, Table S6). Ordinal numbers of the vertebrae associated with the last dorsal fin spine (VtPtLDs) are from this study (see Suppl. Data 3, Table S9). Values in bold indicate those characters of †*Om*. *kabchorensis* and the extant tribes that show overlap.

The majority of the extant species of the haplotilapiine tribes display the standard configuration of five tubules on the lacrimal bone^14,58^. With respect to the EAR tribes, including different lineages of the Haplochromini such as the “*Pseudocrenilabrus* Group”, Altner *et al*.^37^ showed that only four tubules on the lacrimal, as is the case in †*Om*. *kabchorensis*, can occur in species of the Ectodini, Trematocarini, Cyprichromini, Lamprologini, and “*Pseudocrenilabrus* Group”. Regarding the non-EAR tribes, we compiled the possible number of tubules on the lacrimal based on our comparative material and a thorough literature survey (see Suppl. Data 3, Table S6 for details of references). This reveals that the character “four tubules on the lacrimal” occurs among the non-EAR tribes only in the Oreochromini (Fig. 9). Furthermore, we used the comparative dataset as well as the literature presented in the Suppl. Data 3, Table S6 to assemble relevant hard-part-related characters for (i) those EAR tribes that may bear four tubules on the lacrimal and (ii) all non-EAR tribes; the characters include count of total vertebrae, ordinal number of vertebra associated with the pterygiophore of the last dorsal fin spine, dorsal and anal fin formulas, scale type, number of lateral line segments, and supraneural bones. Taking all information together, †*Om*. *kabchorensis* gen. et sp. nov. shows a 100% fit of its fossilised characters with the “*Pseudocrenilabrus* Group” among the EAR tribes and with the Oreochromini among the non-EAR tribes (Fig. 9). Accordingly, we consider these two as candidate tribes or lineages to which †*Om*. *kabchorensis* gen. et sp. nov. might belong, and inspected their further characters.

### Assignment to the *Pseudocrenilabrus* Group?

The extant genus *Pseudocrenilabrus* comprises four described species, which inhabit riverine and lacustrine environments in North, East, Central, and South Africa^36,83,84^. Three of these species, *P*. *multicolor*, *P*. *nicholsi*, and *P*. *philander* form the monophyletic “*Pseudocrenilabrus* Group” within the Haplochromini^36^. The phylogenetic position of the recently described *P*. *pyrrhocaudalis* within this clade has not yet been studied^84^. The four species of *Pseudocrenilabrus* are characterised by the following traits^10,83–87^: number of tubules on the lacrimal bone is four, less frequently five, rarely three or six; caudal-fin shape is rounded or subtruncate; anal fin in adult males bears single orange or red coloured, non-ocellate spot or blotch at its posterior margin. Four tubules on the lacrimal were also present in our comparative material (three specimens each of *P*. *nicholsi* and *P*. *philander*). Hence, †*Om*. *kabchorensis* gen. et sp. nov. shares the presence of four lacrimal tubules with *Pseudocrenilabrus*. The diagnostic value of caudal fin shape is debatable, but it is obvious that †*Om*. *kabchorensis* gen. et sp. nov. has a slightly emarginate caudal fin (Fig. 5b3), vs. rounded or subtruncate in *Pseudocrenilabrus*.

**Figure 10:**
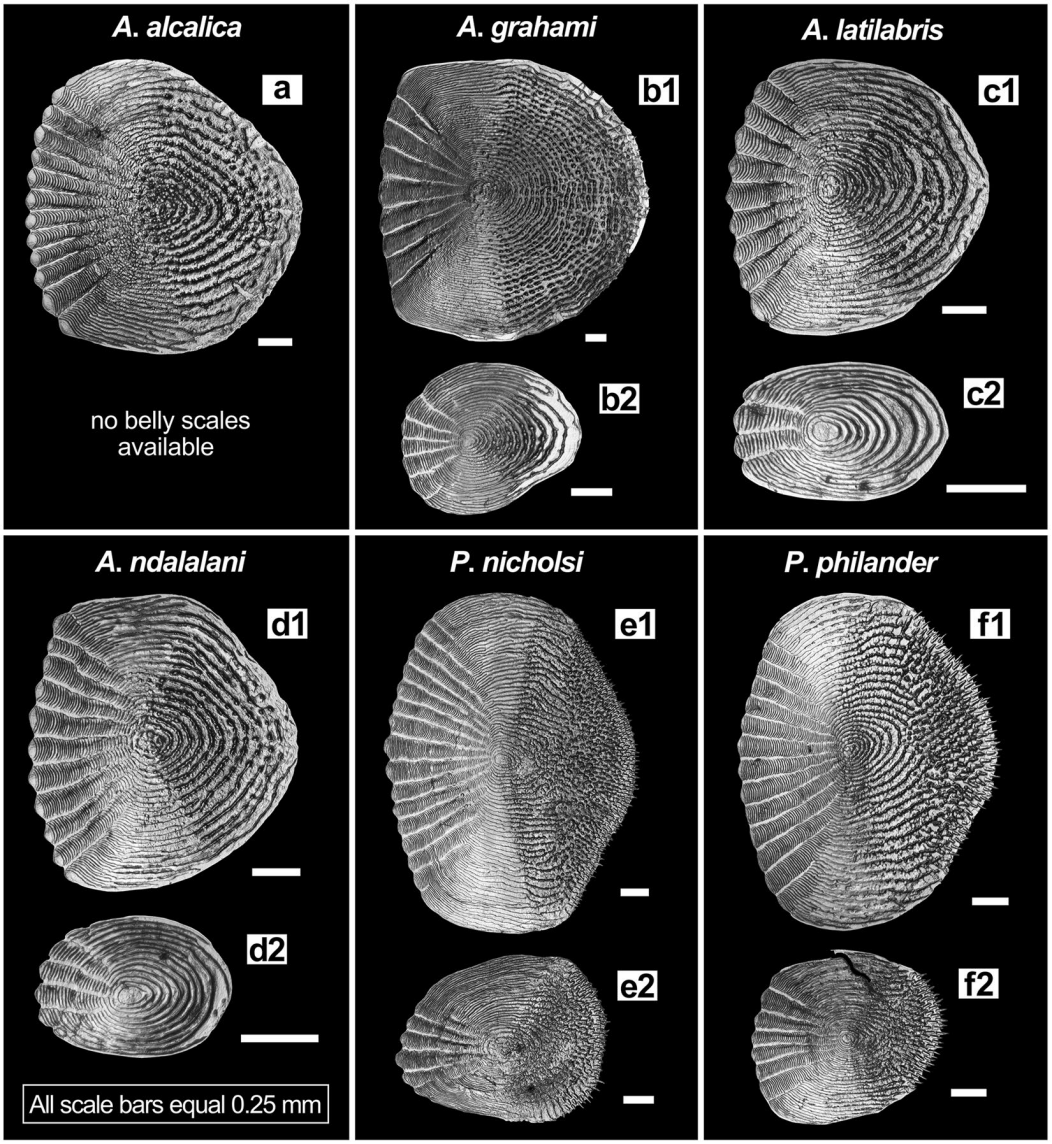
Flank and belly scales from the recent species of *Alcolapia* and *Pseudocrenilabrus* studied here. Scales are shown from the same specimen for each species, except in the case of *A*. *ndalalani*, for which flank and belly scales were obtained from different specimens. All scales are from the left body side, except for the flank scales of *A*. *alcalica* and *P*. *philander* (these scales are mirrored for better comparison). (**a**) ZSM 041072_2, 59.8 mm SL; (**b1–2**) ZSM 025618_2, 90.4 mm SL; (**c1–2**) ZSM 040995_1, 38.7 mm SL; (**d1–2**) ZSM 041055_3, 36.8 mm SL; ZSM 041055_2, 38.1 mm SL; (**e1–2**) ZSM 041575, 60.3 mm SL; (**f1–2**) ZSM 041143_5964, 43.4 mm SL.

To examine further similarities or differences between *Pseudocrenilabrus* and †*Om*. *kabchorensis* gen. et sp. nov. we determined scale width-to-length ratios and relative scale sizes (in % of SL and BL) based on 12 ethanol-preserved specimens of *Pseudocrenilabrus*. The results indicate that flank scales are generally ovate in *Pseudocrenilabrus* (Fig. 10e,f), with a width/length ratio mostly between 1.2–1.3 (Suppl. Data 3, Table S10). In contrast, the fossil taxon has round flank scales (Fig. 4a6), and their width/length ratio is 1.0 (Suppl. Data 3, Table S10). In addition, in all investigated species of *Pseudocrenilabrus* flank scales are larger than in the fossil, with lengths differing by a factor of 1.6–2.0 and 1.4–1.8 (in % of SL and BL, respectively), and widths by a factor of 1.7–2.6 and 1.5–2.4 (in % of SL and BL, respectively). The relative sizes of the belly scales (in % of BL) of *Pseudocrenilabrus* also surpass those of †*Om*. *kabchorensis* gen. et sp. nov. by factors of 2.6–2.8 in length and 3.4–3.9 in width (Suppl. Data 3, Table S11). The marked differences in scale width/length ratios and relative scale sizes do not support the assignment of †*Om*. *kabchorensis* gen. et sp. nov. to the “*Pseudocrenilabrus* Group”.

### Assignment to the Oreochromini?

Ten genera are currently included in the tribe Oreochromini (ref.^60^; Fig. 1). As for the species of *Pseudocrenilabrus*, we examined flank scale width/length ratios and relative flank scale sizes (in % of SL and BL) in 34 ethanol-preserved species of all ten oreochromine genera (63 specimens; Suppl. Data 3, Table S2). Generally, flank scales of the Oreochromini studied can be characterised as moderately rounded, but ovate flank scales, as in *Pseudocrenilabrus*, also occur (in *Danakilia*, *Iranocichla*, *Pungu*, *Tristramella*, and some species of *Oreochromis* and *Sarotherodon*). The same round flank scales (with a width/length ratio of 1.0) as in †*Om*. *kabchorensis* appear in two species of *Alcolapia* (*A*. *grahami*, *A*. *latilabris*) (Suppl. Data 3, Table S12). The relative flank-scale sizes of the Oreochromini studied are generally larger than those of the fossil (as also observed in the *Pseudocrenilabrus* species). Flank-scale lengths (in % of SL and BL) exceed the respective values for the fossil by factors of 1.5–2.3 and 1.3–2.2; flank-scale widths (in % of SL and BL) by factors of 1.6–2.9 and 1.4–2.8 (Suppl. Data 3, Table S12). However, one extant species, *Iranocichla hormuzensis*, has the same (or almost the same) relative flank-scale lengths as †*Om*. *kabchorensis* gen. et sp. nov. (Suppl. Data 3, Table S12). Based on our “best-fit approach”, the identification of two oreochromine species (*Alcolapia*) with the rounded scales seen in the fossil, and at least one oreochromine species (*Iranocichla*) with the same small relative flank-scale lengths as the fossil argues for an assignment of †*Om*. *kabchorensis* gen. et sp. nov. to the Oreochromini rather than the “*Pseudocrenilabrus* Group”.

### Tentative placement of †*Oreochromimos* gen. nov. within the Oreochromini

**Figure 11:**
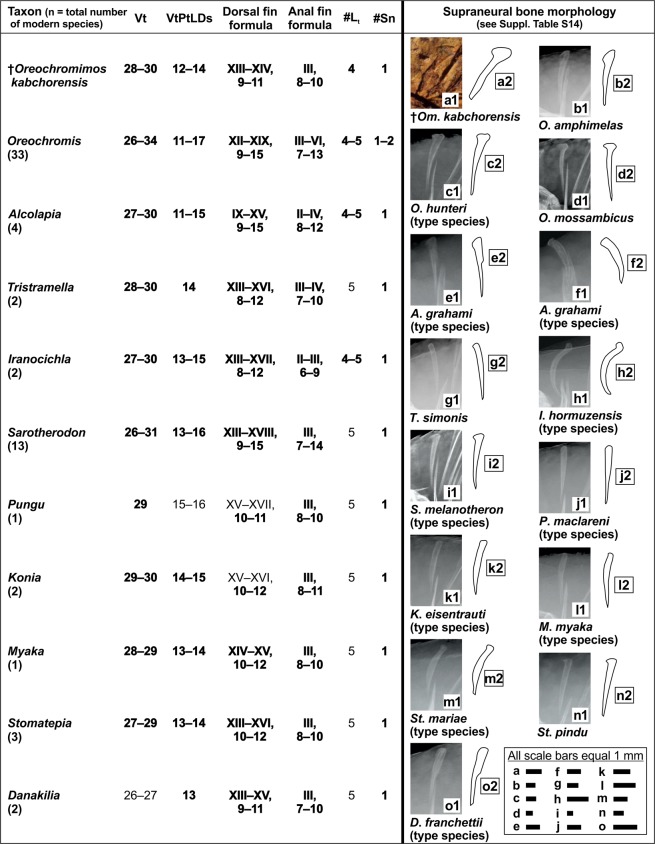
Morphological characters (ranges) of all modern species of the Oreochromini and †*Oreochromimos kabchorensis* gen. et sp. nov., and supraneural bone morphology (left lateral view). Total numbers of species for each genus were compiled from^25,60,111–115^. Supraneural bone morphology and the ordinal number of the vertebra associated with the last dorsal fin spine (VtPtLDs) are from this study. Numbers of total vertebrae (Vt, including urostyle), dorsal/anal fin formulas, and numbers of lacrimal tubules (#L_t_) and supraneural bones (#Sn) are from this study (Suppl. Data 3, Table S9) and from the literature (see Suppl. Data 3, Table S7 for references). Values in bold indicate those characters of †*Om*. *kabchorensis* and the extant genera that show overlap. Pictures of supraneural bones are based on the following specimens: (**a1–2**) OCO-2c-1a(1), holotype; (**b1–2**) ZSM 040956_1; (**c1–2**) BMNH 1952.2.26.53-72_3; (**d1–2**) ZSM 041407; (**e1–2**), (**f1–2**) ZSM 025618_1, ZSM 025618_3; (**g1–2**) ZSM 040017_1; (**h1–2**) SBF 030311_(1); (**i1–2**) BMNH 1981.8.17.54-74_2; (**j1–2**) ZSM 029851_4; (**k1–2**) ZSM 029834_(4); (**l1–2**) ZSM 029844_(4); (**m1–2**) ZSM 029836_3; (**n1–2**) ZSM 029839_3; (**o1–2**) MRAC-164730-732_(720).

**Figure 12:**
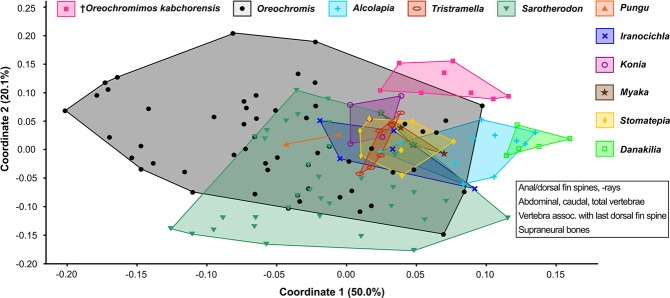
Principal coordinates analysis (PCoA) scatter plot based on eight meristic characters and the number of supraneurals from all modern genera of the Oreochromini (42 species, 181 specimens) and from †*Oreochromimos kabchorensis* gen. et sp. nov. See Suppl. Data 3, Table S9 for raw data.

The question now arises whether the fossil taxon can be assigned to any of the extant oreochromine genera. As we did before at the level of tribes, we used the comparative dataset as well as the literature to assemble the skeletal characters for all known extant oreochromine species. We assembled total vertebrae counts, the ordinal number of the vertebra associated with the pterygiophore of the last dorsal fin spine, dorsal and anal fin formulas, the number of tubules on the lacrimal, and the number of supraneural bones (Fig. 11; for references see Suppl. Data 3, Table S7). The analysis reveals that †*Om*. *kabchorensis* gen. et sp. nov. shares all aforementioned morphological traits with *Oreochromis*, *Alcolapia*, and *Iranocichla* (Fig. 11). Furthermore, because the fossil displays a club-shaped supraneural bone (Fig. 3a3), we asked whether the shape of the supraneural bone might be taxonomically significant. We used the type species of all ten oreochromine genera (except for *Tristramella*) plus 32 other species for this approach (Fig. 11, see Suppl. Data 3, Table S5 for details of specimens used). The supraneural bones of two species of *Oreochromis* (*O*. *chungruruensis*, *O*. *mossambicus*) and one species of *Sarotherodon* (*S*. *linnellii*) were found to show similarity to the supraneural of the new fossil taxon in being relatively straight and distinctly expanded at the distal end. The supraneural bone of *Alcolapia* shows some variation, it can be straight or slightly curved, and a thickened flange at the distal end is present in some specimens of *A*. *alcalica* and *A*. *grahami*. The supraneural of *Iranocichla* is unique due to its strongly bent shape, while the shape of the supraneural in the remainder of our sample of extant species is relatively homogeneous, i.e. straight with at most a very weakly expanded distal head (Suppl. Data 3, Table 14). Thus, the character combination of the new fossil taxon appears most similar to *Oreochromis* and *Alcolapia*. It differs from *Sarotherodon* in the number of lacrimal tubules (4 vs. 5) and from *Iranocichla* with respect to the shape of the supraneural bone (straight vs. bent). An assignment to *Oreochromis* appears to be supported by a multivariate analysis (PCoA) of eight meristic characters and the number of supraneurals: the fossil specimens partially overlap with those of *Oreochromis*, but are relatively distant from *Alcolapia* (Fig. 12). On the other hand, round flank scales with a scale width/length ratio of 1.0, as seen in †*Om*. *kabchorensis* gen. et sp. nov. are found exclusively within *Alcolapia* (see above and Suppl. Data 3, Table S12).

### Assignment to *Alcolapia*?

Trewavas^28^ originally erected *Alcolapia* as a subgenus of *Oreochromis*, but *Alcolapia* has been considered as a distinct genus of the Oreochromini since Seegers *et al*.^88^. The recent study of Ford *et al*.^89^ resurrects the interpretation of Trewavas^28^. *Alcolapia* contains four extant species (Suppl. Data 3, Table S2). One of the diagnostic characters of *Alcolapia* refers to the belly scales, which are described as “very small” in comparison to the flank scales^28^. Additional details were provided by Seegers & Tichy^29^ who reported “small to minute” belly scales for *A*. *alcalica*, and “small” belly scales for the remaining three species. Since “small”, “very small”, and “minute” are subjective terms, we have re-investigated the belly scales of *Alcolapia* (Fig. 10a–d; Suppl. Data 3, Table S13). Our findings show that relative belly-scale sizes (means in % of body length) are distinctive at species level, but always greater than in †*Om*. *kabchorensis* gen. et sp. nov. (Suppl. Data 3, Table S13). Accordingly, the belly scales of †*Om*. *kabchorensis* would correspond to “very small” (*sensu* Trewavas^28^) or “minute” (*sensu* Seegers & Tichy^29^). However, the presence of very small belly scales is not a character that is exclusive to *Alcolapia*, because “very small” belly scales have also been described for some species of *Oreochromis*^28^. A further diagnostic character of *Alcolapia* that can be assessed in the fossil is the lacrimal depth, which is “19–24% length of head at 95–130 mm SL” according to Trewavas^28^. This is clearly larger than that seen in the new fossil taxon, which has a lacrimal depth of 12% in relation to the head length in the holotype, which has a standard length of 109 mm. As a result, although similarities are present, an assignment of the new fossil to *Alcolapia* is not conclusively indicated, which is consistent with the result of the PCoA (see above and Fig. 12).

### Assignment to *Oreochromis*?

In their analysis of osteological differences between *Oreochromis*, *Coptodon*, and *Sarotherodon*, Murray & Stewart^63^ described six characters diagnostic for *Oreochromis*: (i) supraoccipital crest with enlarged posterior tip, recognisable in dorsal view, (ii) opercular bone with posterodorsal excavation, (iii) anteroventral flange of hyomandibula convex, (iv) supraneural bone sharply angled and with distally thickened end, (v) upper process of posttemporal straight, but rounded anteriorly, and (vi) cleithrum with an acute notch in the posteroventral edge of the dorsal plate. The preservation of the fossil specimens allows comparisons of the opercular bone, supraneural bone, and cleithrum. While the shape of the supraneural is the same in the fossil and *Oreochromis*, the posterodorsal margin of the opercle in our new fossil taxon does not bear an depression and the cleithrum does not have any notch on the posteroventral edge of the dorsal plate (Fig. 3a2).

According to Trewavas^28^, species of *Oreochromis* at a standard length of 100–200 mm possess a lacrimal bone depth comprising 18–29% of the head length. This is similar to the proportion reported for *Alcolapia* (see above), but clearly larger than that seen in †*Om*. *kabchorensis* gen. et sp. nov. There is only one species of *Oreochromis* (*O*. *amphimelas*), which shows a lacrimal depth very similar to that of our fossil (12.5–17% of head length at 82–270 mm SL; see Trewavas^28^). In summary, when all similarities and dissimilarities between †*Om*. *kabchorensis* and the extant oreochromine genera are considered, it can be assumed that the fossil taxon shows a very close affinity to both *Oreochromis* and *Alcolapia*, but differs from each based on its particular combination of characters (Table 1).

**Table 1:** Character combination of †*Oreochromimos* gen. nov. in comparison to the extant genera *Alcolapia* and *Oreochromis*. Abbreviation: ?, not known.

	Four tubules on lacrimal	Total vertebrae count	Position of vertebra associated with pterygiophore of last dorsal fin spine	Dorsal fin formula	Anal fin formula	Shape of supraneural bone	Relative flank scale length	Very small to minute belly scales	Width/Length of flank scale is 1.0	Lacrimal depth	Notch on cleithrum
†*Oreochromimos*	+	+	+	+	+	+	+	+	+	+	−
*Alcolapia*	+	+	+	+	+	(+)	−	+	+	−	?
*Oreochromis*	+	+	+	+	+	+	−	+	−	(+)	+

### Affinities with previously described fossil Oreochromini

**Figure 13:**
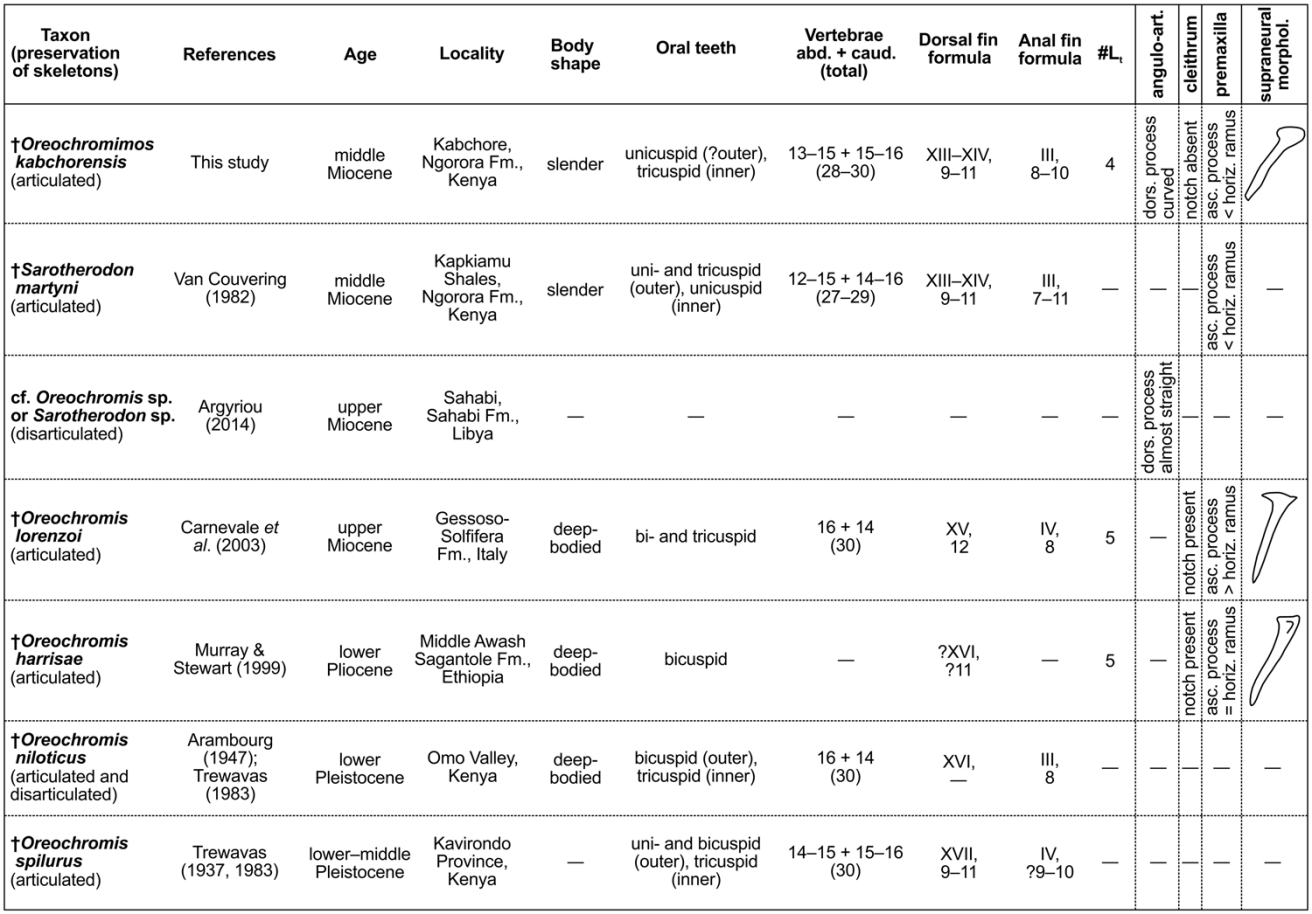
Summary of all previously described African and European fossil cichlids of the tribe Oreochromini, and morphological characters differentiating them from †*Oreochromimos kabchorensis* gen. et sp. nov. Supraneural bone morphology of †*Oreochromis lorenzoi* and †*O*. *harrisae* redrawn from Fig. 3a in Carnevale *et al*.^38^ and Fig. 5d in Murray & Stewart^63^ (mirrored). Abbreviations: —, unknown character; #L_t_, number of lacrimal tubules.

†*Oreochromis lorenzoi* from the upper Miocene (ca. 6 Ma) of Italy, †*O*. *harrisae* from the lower Pliocene (ca. 4.4–4.3 Ma) of Ethiopia, and †*O*. *niloticus* and †*O*. *spilurus* from the Pleistocene of Kenya can be clearly separated from our new fossil taxon with respect to their deep body shapes, their oral dentition, and a slightly higher number of dorsal fin spines (see Fig. 13 for details). Furthermore, †*Om*. *kabchorensis* gen. et sp. nov. differs from †*O*. *lorenzoi* and †*O*. *harrisae* in having four lacrimal tubules (vs. five), in the absence of an acute notch (*sensu* Murray & Stewart^63^) on the cleithrum (vs. present), and in possessing a premaxillary ascending process that is shorter than the horizontal ramus (vs. slightly longer or about as long), and a supraneural bone characterised by a prominent expansion at its distal end (vs. no expansion) (Fig. 13).

Five fossil species assigned to the Oreochromini have been described in previous studies based on articulated skeletons (Fig. 13): †*Oreochromis lorenzoi*, †*O*. *harrisae*, †*O*. *niloticus* (valid name for †*Tilapia crassispina* according to Trewavas^28^), †*O*. *spilurus* (valid name for †*Tilapia nigra* according to Trewavas^28^), and †*Sarotherodon martyni*. Further disarticulated fossil remains of the Oreochromini have been described as “cf. *Oreochromis* sp. or *Sarotherodon* sp.” by Argyriou^90^.

The fossil remains of “cf. *Oreochromis* sp. or *Sarotherodon* sp.” are from the upper Miocene (ca. 7 Ma) of Libya and comprise a single angulo-articular bone and an isolated second vertebra (see fig. 3.18A–B in Argyriou^90^). The former can be clearly distinguished from that seen in †*Om*. *kabchorensis* gen. et sp. nov., because its dorsal process is almost straight (vs. curved, see Fig. 3a2). It is not possible to compare the second vertebra with that of †*Om*. *kabchorensis*, because this element is not well preserved in the latter.

†*Sarotherodon martyni* is particularly noteworthy because it was found not far from Kabchore (the type locality of †*Om*. *kabchorensis*) in the “Kapkiamu Shales”^47^. While Kabchore is part of the lower segment of “Member C” of the Ngorora Fm. with an age of ca. 12.5 Ma, the “Kapkiamu Shales” represent a younger segment of “Member C”, with an age of ca. 12 Ma^62^. †*Oreochromimos kabchorensis* and †*S*. *martyni* display similar body and head proportions, fin counts, and numbers of abdominal and caudal vertebrae (Fig. 13). In addition, they share the presence of a single supraneural bone (shape unknown in †*S*. *martyni*), and tiny belly scales together with large flank scales. However, there are also some important differences. According to Van Couvering’s^47^ original description, the inner oral dentition of †*S*. *martyni* is comprised entirely of unicuspid teeth, whereas tricuspid inner teeth occur in †*Om*. *kabchorensis* (Fig. 5a2). Moreover, the shape of the premaxillary bone clearly differentiates the two species (see pl. 115 in Van Couvering^91^ and Fig. 3a2): the anterior end of the premaxillary bone is protruding and rounded in †*Om*. *kabchorensis* (vs. not protruding, but slightly pointed in †*S*. *martyni*), and the anterior tip of the horizontal ramus is strongly bent (vs. very slightly bent). Further differences concern the opercle. Although it is not completely preserved in †*Om*. *kabchorensis*, it clearly shows an almost straight anterior and oblique posteroventral margin, whereas the posteroventral margin has a marked depression in †*S*. *martyni*. Another difference relates to the morphology of the basipterygium, whose ventral and posteroventral margins appear less rounded in †*Om*. *kabchorensis* than in †*S*. *martyni* (see Fig. 3a4, see also pl. 115 in Van Couvering^91^). A further important distinction relates to the extension of the dorsal and anal fin rays, which – unlike the case in †*S*. *martyni* – do not reach the origin of the caudal fin or beyond in †*Om*. *kabchorensis* (Figs 3a4 and 5a1,b1). Finally, there are slight differences in the lateral line system. The lateral line pattern of †*S*. *martyni* is clearly recognisable from the photo provided in pl. 115 in Van Couvering^91^. This shows that †*S*. *martyni* has 9–10 tubular scales in the posterior lateral-line segment (vs. >10 in †*Om*. *kabchorensis*), which is running immediately adjacent to the vertebral column (vs. slightly below the vertebral column in †*Om*. *kabchorensis*), and the posterior trunk segment is simply an extension of the anterior segment, without any overlap (vs. overlap by several scale rows in †*Om*. *kabchorensis*; see Fig. 4a5).

### Affinities with previously described non-oreochromine fossils from the Ngorora Formation

Three further fossil cichlid species have recently been described on the basis of articulated skeletons from the same formation (Ngorora Formation) and region (Tugen Hills, Central Kenya) in which †*Om*. *kabchorensis* was discovered. They are represented by two species of †*Rebekkachromis* (†*R*. *ngororus*, †*R*. *kiptalami*), and by †*Tugenchromis pickfordi* (see^37,39^). According to the lithostratigraphy of Rasmussen *et al*.^62^, these three species come from younger strata of the Ngorora Fm. than †*Om*. *kabchorensis*, i.e. from “Member D” (ca. 11 Ma) in the case of †*R*. *ngororus* and †*R*. *kiptalami*, and from “Member E” (ca. 10–9 Ma) in the case of †*T*. *pickfordi*.

†*Rebekkachromis* has been referred to as a putative member of the tribe Etiini, and is currently considered as the earliest representative of the haplotilapiines^39^. It can be clearly distinguished from †*Om*. *kabchorensis* because it possesses two supraneural bones. †*Tugenchromis pickfordi* has been tentatively placed within the tribes of the EAR^37^. It is characterised by a lacrimal bone with six tubules (four in †*Om*. *kabchorensis*) and a tripartite lateral line on the trunk (bipartite in †*Om*. *kabchorensis*). Hence, †*Om*. *kabchorensis* clearly differs from both †*R*. *ngororus* and †*R*. *kiptalami*, and also from †*T*. *pickfordi*.

## Conclusions

The middle Miocene site Kabchore constitutes a unique archive with high preservation quality for the exploration of fossil cichlids within the Tugen Hills (Ngorora Formation, Central Kenya Rift). Our new fossil taxon †*Om*. *kabchorensis* gen. et sp. nov. and the recently reported †*Rebekkachromi*s from younger strata of the same formation and region, represent the only two reliably identified fossil haplotilapiines found so far.

Most of the previously described fossil cichlids from Africa and the Middle East could not be analysed in sufficient detail to enable their systematic position to be determined (e.g.^47,92^), owing to poor preservation and lack of information regarding the ranges of various meristic and osteological characters within modern cichlid tribes. In this study, we present an expanded version of the dataset assembled by Altner *et al*.^37^. This now incorporates results from additional comparative material and data derived from all available literature, including meristic counts, scale types, and numbers of lateral line segments, tubules on the lacrimal, and supraneural bones for all known species of all non-EAR tribes. On this basis, we propose to assign †*Om*. *kabchorensis* gen. et sp. nov. to the tribe Oreochromini. It shows similarities to the extant genera *Oreochromis* and *Alcolapia*, and represents the oldest known species of the Oreochromini. It thus provides a new calibration constraint for estimates of divergence times of the East African cichlids because the previously used “oldest” species of the Oreochromini, i.e. †*O*. *lorenzoi* is of upper Miocene age (6 Ma), and hence significantly younger than our new find.

## Materials and Methods

### Institutional abbreviations

See Suppl. Data 3, Table S1.

### Comparative material from extant cichlids

We used ethanol- or formalin-preserved specimens of the cichlid species represented in the Bavarian State Collection of Zoology, Munich (Germany) to generate X-ray images using an UltraFocus Digital Radiography System (Faxitron LX-60, Faxitron Bioptics LLC). The X-ray dataset comprises 70 cichlid species representing 20 genera (322 specimens), covering the nine haplotilapiine tribes that do not belong to the EAR (Fig. 1). It includes all known species of the Etiini, Coelotilapiini, Pelmatotilapiini, and Tilapiini, all known species except *Heterotilapia cessiana* of the Heterotilapiini, and all known species except *Tilapia busumana* of the Gobiocichlini. For the Oreochromini, Steatocranini, and Coptodonini the X-ray dataset comprises all present-day genera and important phylogenetic lineages, but not all species. For details see Suppl. Data 3, Table S1. Based on the X-rays, 9 meristic characters were counted, namely (i–iv) numbers of dorsal and anal fin spines and rays, (v-vii) counts of abdominal, caudal, and total vertebrae including the urostyle, (viii) the number of supraneural bones, and (ix) the ordinal number of the vertebra (counted anteriorly to posteriorly) that is associated with the pterygiophore of the last dorsal fin spine. All data is provided in Suppl. Data 3, Table S9.

**Figure 14:**
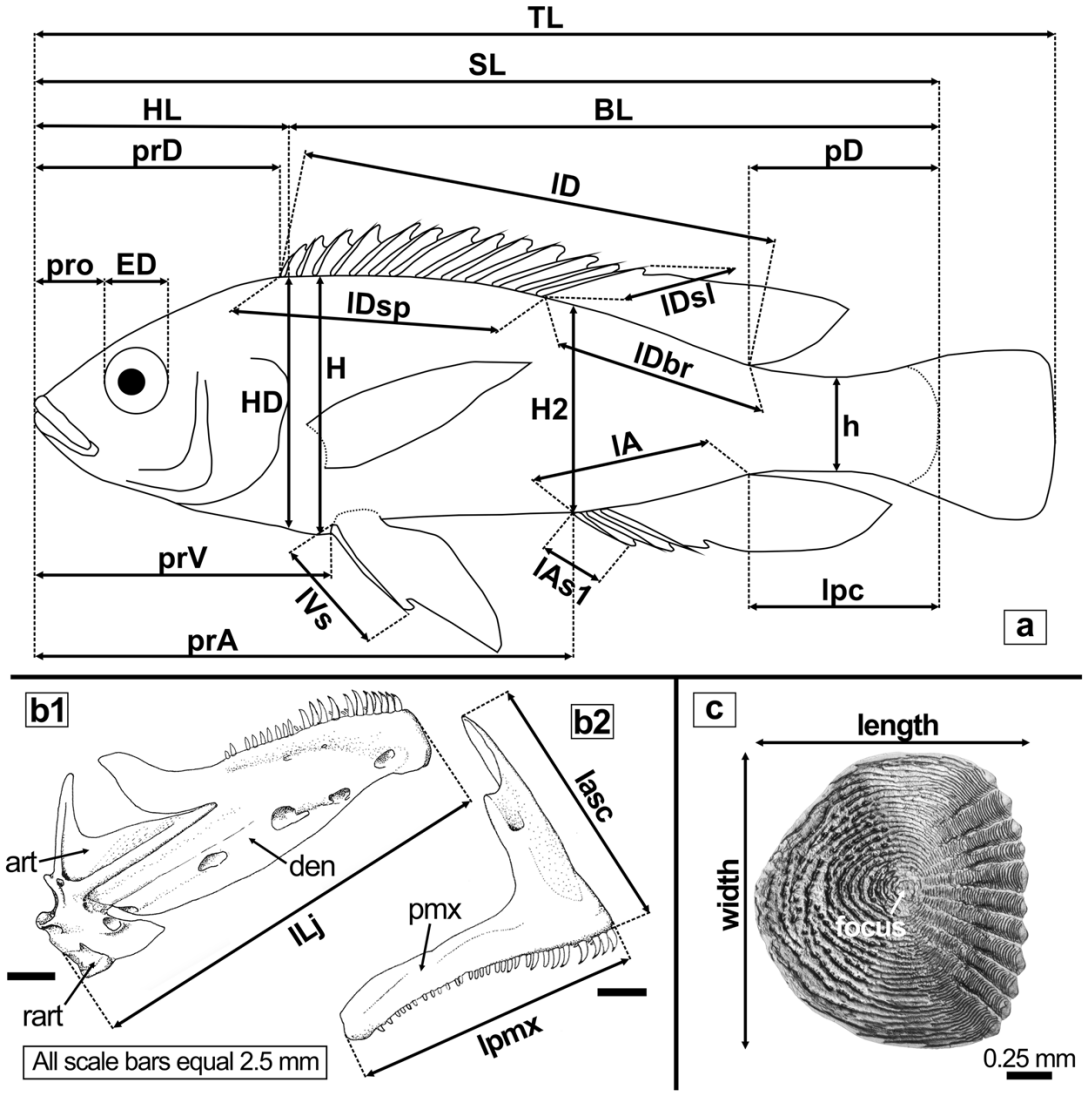
Morphometric measurements and scale size analyses (double arrows) conducted for this study. (**a**) Schematic drawing of a generalized cichlid depicting the various head-, body-, and fin-related measurements used to characterise specimens. (**b1–2**) Drawings of the lower jaw bone (**b1**) and the upper jaw bone of *Haplochromis vonlinnei* (**b2**) in right lateral view, depicting the measurements taken (reprinted from Figs 3 and 6 in Van Oijen & De Zeeuw^116^, with permission from Naturalis Biodiversity Center). (**c**) Scale surface showing the measurements used to characterise the form of flank and belly scales (right flank scale of *Alcolapia ndalalani*, ZSM 041055_1). Abbreviations: art, angulo-articular; BL, body length; den, dentary; ED, horizontal eye diameter; H, maximum body height; h, minimum body height; H2, maximum body height at origin of anal fin; HD, head depth; HL, head length; lA, length of anal fin base; lAs1, length of first anal fin spine; lasc, length of premaxillary ascending process; lD, length of dorsal fin base; lDbr, length of soft dorsal fin base; lDsl, length of last dorsal fin spine; lDsp, length of spinous dorsal fin base; lLj, length of lower oral jaw; lpc, length of caudal peduncle; lpmx, length of premaxilla; lVs, length of pelvic fin spine; pD, postdorsal distance; pmx, premaxilla; prA, preanal distance; prD, predorsal distance; pro, preorbital distance; prV, prepelvic distance; rart, retro-articular; SL, standard length; TL, total length.

Furthermore, ethanol-preserved specimens of the Oreochromini (34 species) and of *Pseudocrenilabrus* (5 species) were used to extract scales from the belly and the flanks (see Suppl. Data 3, Tables S2, S3 for details on specimens). Only “normal scales”, i.e. with no evidence of alteration regarding the focus and surrounding circuli, were extracted (see^93^). Belly scales were sampled from the left body side between the pelvic fin base and the anal fin origin. Flank scales were sampled from the third or fourth row below the dorsal fin (counted dorsally to ventrally) from the left body side, if flank scales were lacking on the left side, they were removed from the right side. Scales were cleaned following the protocol of Gholami *et al*.^93^ and dried overnight between two microscope slides. Generally, 3–6 normal belly scales and 1–4 normal flank scales were prepared for each individual. Length and width of scales were measured (see Fig. 14c) from digital images using ImageJ version 1.51m9 64-bit^94^ (accuracy ± 0.01 mm) and standardised based on the standard and body length of the respective specimen. Further ethanol-preserved material used for the study of lacrimal morphology included *Alcolapia* (4 species), *Pseudocrenilabrus* (2 species), *Iranocichla hormuzensis*, and *Coelotilapia joka* (see Suppl. Data 3, Table S4 for details on specimens).

### Compilation of oral jaw teeth sizes of extant cichlids

We used published drawings and photos from^28,80,95^ to estimate the maximum crown widths of tricuspid inner and outer teeth of *Oreochromis*, *Alcolapia*, *Sarotherodon*, and *Danakilia* (18 species, 27 specimens, see Suppl. Data 3, Table S16).

### Fossil specimens

The fossil material consists of 8 articulated specimens preserved in lateral view, 4 of which were complete. Where necessary, remnants of sediment were mechanically removed with the aid of an engraving pen (powered by compressed air) and a dissecting needle; fragile structures were fixed by using a mixture of Mowilith (a polyvinyl acetate) and acetone. All fossils are currently housed in the Department of Earth and Environmental Sciences, Ludwig-Maximilians-Universität München, Munich, but will be transferred to Kipsaraman, Kenya, upon completion of the new Baringo County Geopark. Inventory numbers are OCO-2a-10a, b*; OCO-2c-1a, b(1)*; OCO-2c-4a, b*; OCO-2c-13a, b*; OCO-2a-5; OCO-2a-13(1); OCO-2c-1a, b(3); OCO-2c-5a, b(1). OCO stands for the Orrorin Community Organisation, which is the legal owner of this material. The labels 2a, c refer to the site. The last number after the hyphen refers to the specimen, “a, b” indicates that both part and counterpart are preserved. A number in brackets at the end of the inventory number identifies the individual specimen when more than one was preserved on the same slab. The star symbol (*) is used for complete specimens.

The fossil specimens were studied and photographed with a Leica M165 FC stereomicroscope that was fitted with a Leica DFC450 digital camera. Digital images were processed in Adobe Photoshop CS6 version 13.0 64-bit (©1990–2012 Adobe Systems Incorporated). Interpretation of osteological characters, scale size and morphology, and dentition followed^14,35,47,51–54,58,64,73,74,76,96–98^, unless otherwise mentioned. Meristic counts included the same nine characters as calculated for the extant X-rayed specimens (see above). Head-, body-, and fin-related linear measurements of the fossil specimens, as shown in Fig. 14a, followed the methods and terminology of Barel *et al*.^96^ and Altner *et al*. (see Fig. 1C in Altner *et al*.^37^). Body length was defined according to Van Couvering^47^ and is the distance from the posterior margin of the opercle to the posterior margin of the hypural plate; this length is particularly useful for fossil specimens that are incomplete. Depth measurement of the lacrimal bone was conducted according to Trewavas^28^. Length measurement of the upper and lower oral jaw bones (see Fig. 14b) were taken according to^28,68^. Measurements of scales (see Fig. 14c) and oral tooth width were taken as well. While small elements (lacrimal, scales, teeth) were measured based on digital images by using ImageJ, all other measurements were conducted with an electronic digital vernier calliper to the nearest 0.1 mm. Meristic counts, raw head-, body-, and fin-related measurements and the respective standardised values are presented in Suppl. Data 3, Table S15, scale measurements are provided in Suppl. Data 3, Tables S10–S13.

### Statistics

A principal coordinates analysis (PCoA) was run using PAST (Paleontological Statistics; ref.^99^) version 3.18, in order to visualize similarities between the meristic traits and the modern lineages of the Oreochromini (42 species; see Suppl. Data 3, Table S9). The advantage of a PCoA compared to the principal components analysis (PCA) is that it generates more reliable results if values are missing^100,101^, as was the case in some of our fossil specimens.

### Phylogenetic analyses

Standard categorical matrices were compiled in Mesquite 3.51^102^, using the parsimonious tree model with all character states unordered (available in Nexus format, Suppl. Data 1, 2). In the matrix based on Stiassny^14^, we used the “generalized percomorph outgroup” (see page 7 in Stiassny^14^, see also Stiassny^69,103^); codings for this outgroup were not explicitly listed in Stiassny^14^, but could be compiled from her text (see Suppl. Data 3, Table S17 for details). In the matrix based on Takahashi^35^, we used only one of his five outgroup species – *Tylochromis polylepis* – because this species was the most basal in his tree (see Fig. 12 in Takahashi^35^; Suppl. Data 2). Stiassny^14^ had coded 28 morphological characters as two-state characters of equal weight for 18 ingroups, including *Heterochromis*. In our analysis, we did not consider *Heterochromis*, as this taxon has displayed an unstable position in other morphology-based phylogenetic analyses (see e.g.^53,58^). The new fossil was added by inserting character states for six (out of 28) characters (the respective states are given in parentheses), i.e. characters 5(0), 10(0), 11(0), 17(0), 25(1), 27(1). These characters refer to the morphology of the infraorbital bones and urohyal, the lateral line system of the neurocranium, the opercular apparatus, and the numbers of vertebrae and supraneural bones (see Suppl. Data 3, Table S17 for details). Unknown characters/states were indicated with “?”. Takahashi^35^ had originally coded 37 morphological characters for 67 ingroup species from Lake Tanganyika and used up to six states for a particular character (all states were of equal weight). The only change we made was to split Takahashi’s six-state character 1 (“infraorbitals”) into five newly defined two- to three-state characters by using the data provided in Takahashi^54^; this results in a total of 41 characters. Furthermore, we updated the taxonomy of the species used in Takahashi^35^ based on the Catalog of Fishes^104^. The attribution of each species to one of the haplotilapiine lineages was taken from^34,36,60,65,105,106^. Accordingly, the data matrix of Takahashi^35,54^ includes members of the Oreochromini and Coptodonini and of all 12 lineages of the EAR (Haplochromini and Tropheini are counted as one lineage) (Suppl. Data 2). To this matrix we added †*Oreochromimos kabchorensis* based on 15 (out of 41) characters (respective states given in parentheses): 1(1), 3(1), 4(1), 5(0), 13(1), 17(0), 18(0), 19(1), 23(0), 26(0), 36(0), 37(0), 38(0), 39(0), 41(0); these characters relate to the morphology of the infraorbital bones, hyoid arch, and urohyal bone, the shape of the inner oral jaw teeth and caudal fin, the number of anal fin spines and lateral line foramina on the opercular apparatus, scale type and squamation pattern, and the extent of the nuchal hump (see Suppl. Data 3, Table S18 for details). Unknown characters/states are indicated again with “?”.

Phylogenetic analyses were performed under maximum parsimony for each matrix in TNT 1.1 (Willi Hennig Society Edition; ref.^107^), using a combination of “New Technology” search options, i.e. parsimony ratchet, tree-drifting, and tree-fusing. All other settings were left at their defaults. Clade support was estimated using standard bootstrapping (1000 replicates, absolute frequency values). Clades with bootstrap values ≥ 70% were considered significant and strongly supported according to Hillis & Bull^108^. Phylogenetic trees were visualized and edited in FigTree 1.4.3^109^.

The Fig. 2 is reprinted from Palaeogeography, Palaeoclimatology, Palaeoecology, volume 279, Kiage, L. M. & Liu, K.-b., Palynological evidence of climate change and land degradation in the Lake Baringo area, Kenya, East Africa, since AD 1650, p. 60–72. Copyright (2009), with permission from Elsevier.

The Figure 14b1,b2 is reprinted from Zoologische Mededelingen, volume 82, Van Oijen, M. J. P. & De Zeeuw, M. P., Haplochromis vonlinnei spec. nov., a piscivorous haplochromine cichlid (Teleostei, Perciformes) from the Mwanza Gulf area of Lake Victoria, Tanzania, p. 167–175. Copyright (2008), with permission from Naturalis Biodiversity Center.

The photographs in Figs 3a1,a4, 4a1,a4, 5a1,b1,b3 have been taken by M. Schellenberger, employed at the SNSB - Bavarian State Collection of Palaeontology and Geology (BSPG). Copyright (2019), with permission from SNSB - BSPG.

## Supplementary information

Supplementary Data 1 and 2

Supplementary Data 3, Tables S1 - S18

Supplementary Information
