# Supplementary material for: New fossil cichlid from the middle Miocene of East Africa revealed as oldest known member of the Oreochromini
Source: Sci Rep. 2019 Jul 15;9:10198. doi: 10.1038/s41598-019-46392-5 (PMC6629881; doi:10.1038/s41598-019-46392-5)
Supplement: Supplementary file 3 — Supplementary Information [file 41598_2019_46392_MOESM3_ESM.docx]

**New fossil cichlid from the middle Miocene of East Africa revealed as oldest known member of the Oreochromini**

**Stefanie B. R. Penk^1^*****, Melanie Altner^1^, Ulrich K. Schliewen^2,3^, Alexander Cerwenka^4^ & Bettina Reichenbacher^1,2^***

^1^Department of Earth and Environmental Sciences, Ludwig-Maximilians-Universität München, 80333 Munich, Germany

^2^GeoBio-Center, Ludwig-Maximilians-Universität München, 80333 Munich, Germany

^3^Department of Ichthyology, SNSB Bavarian State Collection of Zoology, 81247 Munich, Germany

^4^Section Evertebrata varia, SNSB Bavarian State Collection of Zoology, 81247 Munich, Germany

**Legend for Supplementary Files**

**Supplementary Data 1.** Nexus file used for phylogenetic analysis on the basis of the matrix of Stiassny (1991). See Suppl. Data 3, Table S17 for details of characters and states.

**Supplementary Data 2.** Nexus file used for phylogenetic analysis on the basis of the matrix of Takahashi (2003a, 2003b). See Suppl. Data 3, Table S18 for details of characters and states.

**Supplementary Data 3, Tables S1–S13**

Table S1. Comparative material of extant species used for X-ray analysis and assembly of meristic data. All non-EAR tribes of the haplotilapiines are represented: Etiini (monotypic, 1 species), Oreochromini (all genera, 42 species), Coelotilapiini (monotypic, 1 species), Coptodonini (1 genus, 6 species), Heterotilapiini (1 species (out of 2)), Gobiocichlini (3 genera, 5 species = all known species except *Tilapia busumana*), Pelmatotilapiini (1 genus, 2 species = all known species), Steatocranini (1 genus, 4 species), Tilapiini (3 genera, 8 species = all known species). See Methods for additional details.

Table S2. Comparative material of extant species of the Oreochromini (34 species) and the *Pseudocrenilabrus* Group (5 species) used for flank scale analysis. For institutional abbreviations see Table S1.

Table S3. Comparative material of extant species used for belly scale analysis. For institutional abbreviations see Table S1.

Table S4. Comparative material of extant species of the Oreochromini (5 species), Coelotilapiini (1 species), and the *Pseudocrenilabrus* Group (2 species) used for lacrimal analysis. For institutional abbreviations see Table S1.

Table S5. Comparative material of extant species of the Oreochromini used for supraneural morphology analysis. For institutional abbreviations see Table S1.

Table S6. Literature used to assemble morphological information for all genera and species of the non-EAR tribes of the haplotilapiines and for all genera and species of the EAR tribes Lamprologini, Ectodini, Cyprichromini, Trematocarini, and the *Pseudocrenilabrus* Group (data depicted in Figure 9).

Table S7. Literature used to assemble additional morphological information for the Oreochromini (data depicted in Figure 11).

Table S8. Literature used to assign a given species for which morphological information has been assembled to one of the recognised tribes (data depicted in Figure 9).

Table S9. Meristic data for non-EAR haplotilapiines on the basis of our X-rayed dataset (see Table S1). Data for EAR tribes from Altner *et al*. (2017). For institutional abbreviations see Table S1.

Table S10. Sizes of flank scales and scale width/length ratios of †*Oreochromimos kabchorensis* gen. et sp. nov. and extant species of *Pseudocrenilabrus*. BL, body length (in mm); SL, standard length (in mm); n, number of normal flank scales from left (l) or right (r) body side; SD, standard deviation; —, unknown.

Table S11. Sizes of belly scales in % of body length (BL) of †*Oreochromimos kabchorensis* gen. et sp. nov. and extant species of *Pseudocrenilabrus*. SD, standard deviation.

Table S12. Sizes of flank scales and scale width/length ratios of †*Oreochromimos kabchorensis* gen. et sp. nov. and extant species of Oreochromini. BL, body length (in mm); SL, standard length (in mm); n, number of normal flank scales from left (l) or right (r) body side; SD, standard deviation; —, unknown.

Table S13. Sizes of belly scales in % of body length of †*Oreochromimos kabchorensis* gen. et sp. nov. and extant species of *Alcolapia*. Length and width of belly scales could be measured for specimens of *A*. *grahami*, *A*. *latilabris*, and *A*. *ndalalani*, while no belly scales were preserved in *A*. *alcalica*. BL, body length (in mm); SL, standard length (in mm); n, number of normal belly scales from left (l) or right (r) body side; SD, standard deviation.

Table S14. Supraneural morphology of extant Oreochromini. See Table S5 for specimens used.

Table S15. Measurements (in mm) and counts for holotype OCO-2c-1a, b(1) and paratypes of †*Oreochromimos* *kabchorensis* gen. et sp. nov. Measurements in % of standard and body lengths are added in brackets. —, not detectable.

Table S16. Maximum crown widths of tricuspid oral teeth from specimens of †*Oreochromimos kabchorensis* gen. et sp. nov. (this study) and from extant species of Oreochromini according to literature data (Trewavas, 1962, 1983; Tichy & Seegers, 1999).

Table S17. Character list and states used for phylogenetic analysis (compiled from Stiassny, 1991).

Table S18. Character list and states used for phylogenetic analysis. Characters 1–5: newly compiled based on the information in Takahashi (2003a). Characters 6–41: compiled from Takahashi (2003b).
